# Metabolomic Analysis, Fast Isolation of Phenolic Compounds, and Evaluation of Biological Activities of the Bark From *Weinmannia trichosperma* Cav. (Cunoniaceae)

**DOI:** 10.3389/fphar.2020.00780

**Published:** 2020-05-27

**Authors:** Ruth Barrientos, Carlos Fernández-Galleguillos, Edgar Pastene, Mario Simirgiotis, Javier Romero-Parra, Shakeel Ahmed, Javier Echeverría

**Affiliations:** ^1^ Instituto de Farmacia, Facultad de Ciencias, Universidad Austral de Chile, Valdivia, Chile; ^2^ Laboratorio de Síntesis y Biotransformación de Productos Naturales, Departamento de Ciencias Básicas, Facultad de Ciencias, Universidad del Bío-Bío, Chillán, Chile; ^3^ Departamento de Química Orgánica y Fisicoquímica, Facultad de Ciencias Químicas y Farmacéuticas, Universidad de Chile, Santiago, Chile; ^4^ Departamento de Ciencias del Ambiente, Facultad de Química y Biología, Universidad de Santiago de Chile, Santiago, Chile

**Keywords:** *Weinmannia trichosperma*, tineo, astilbin, traditional medicine, HPLC-MS, centrifugal partition chromatography

## Abstract

*Weinmannia trichosperma* Cav. (Cunoniaceae) (local name, tineo; Mapuche names, madén, mëdehue) is an endemic species of Chile and Argentina used in Mapuche traditional medicine in the treatment of chronic diarrhea, inflammation, and wound healing. This study focused on the isolation, analysis, and characterization of the biological activity of compounds and bark extracts from this plant for the first time. The infusion and tincture of the bark were characterized regarding antioxidant and important enzyme inhibitory activities, phenolics, and flavonoids content and UHPLC-ESI-OT-MS metabolite profiling. Twenty-five metabolites were detected in the medicinal infusion of *W. trichosperma*, three flavonols were isolated: isoastilbin, neoisoastilbin, and neoastilbin ((2*R*,3*S*)-, (2*S*,3*R*)-, and (2*S*,3*S*)-dihydroquercetin 3-*O*-alpha-l-rhamnoside) by countercurrent chromatography, and the isomers were quantified in the bark using a validated analytical HPLC methodology. The antioxidant properties were measured by ABTS, DPPH, FRAP, ORAC, and TEAC methods. The infusion displayed a strong DPPH and ABTS scavenging activity (IC_50_ = 20.58 and 3.070 µg ml^−1^, respectively) while a moderated effect was observed in the FRAP, ORAC, and ABTS assays. The infusion showed a content of phenolic and flavonoid compounds of 442.1 mg GAE g^−1^ and 15.54 mg QE g^−1^, respectively. Furthermore, the infusion showed a good and promissory inhibitory activity (33.80%, 33.12%, and 82.86% for AChE, BuChE, and 5-hLOX, respectively) and isoastilbin (51.70%, 50.10%, and 34.29–80.71% for AChE, BuChE, and 5-hLOX, respectively). The biomolecules identified in this study support the traditional uses of this bark and the potential industrial interest from this Valdivian plant species.

## Introduction

**Figure 1:**
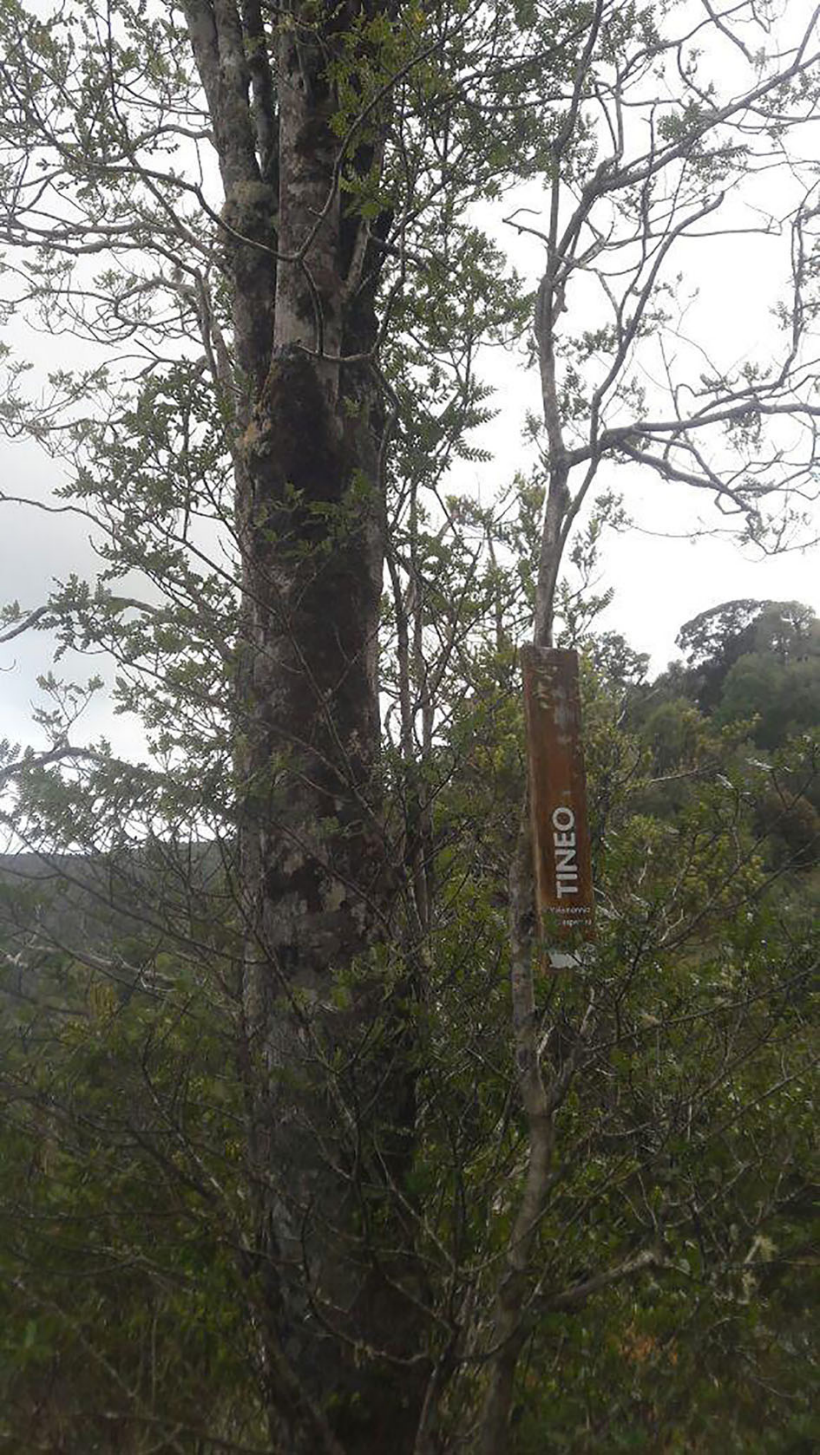
Picture of *W. trichosperma* taken in March 2017 in Parque Oncol, Valdivia.

*Weinmannia trichosperma* Cav. (Cunoniaceae) (local name: tineo, Mapuche name: madén, mëdehue, **Figure 1**) is an evergreen tree endemic of temperate rainforest of Chile and Argentina. In Chile, this plant grows in the regions of Maule, Biobío, Araucanía, Los Ríos, Los Lagos, Aysén, and Magallanes (Rodriguez et al., 2018). The local inhabitants use the bark infusion of *W. trichosperma* to treat chronic diarrhea, and it is used as poultice for application on wounds (Gusinde, 1936; Muñoz et al., 1981; Houghton and Manby, 1985). Previous pharmacological studies of *W. trichosperma* have demonstrated their antimicrobial potential (Mølgaard et al., 2011), and one quantitative phytochemical study showed that the composition of tannins and phenolic compounds of this bark were 19.98% and 4.81%, respectively (Holler et al., 2012). Extracts of natural products are highly complex mixtures of active compounds, and the purification is a challenging task (Skalicka-Woźniak and Garrard, 2014). Among the chemical methods for the study of plants, chromatographic analysis has a fundamental role because it involves numerous advantages, such as its specificity and the possibility of qualitative and quantitative analyses (Kowalska et al., 2007). The interest in endemic traditional plants is important since they represent a huge source very little explored of potential biomolecules that can become potential promising candidates for the study of new drugs of pharmacological interest and support the use of native species in functional foods or nutraceuticals. Ultra-high-performance chromatography (UHPLC) coupled to mass spectrometer (MS) hyphenated devices nowadays offer higher sample throughput with more accurate information per sample. In the case of orbitrap technology (OT), it offers the fast and reliable untargeted information of several different metabolites, such as phospholipids, glycans, phenolic compounds, peptides, pesticides, toxins, and other small bioactive molecules (Simirgiotis et al., 2016; Luna et al., 2018; Deng et al., 2019, Yang et al., 2018a). To our knowledge, there are no reports on chemical characterization, antioxidant, antiinflammatory, or enzyme inhibitory capacities of the phenolic-enriched bark obtained from *W. trichosperma*.

Centrifugal partition chromatography (CPC) (Ito, 2005) is a very versatile technique that has been used for decades as a fractionation technique, and nowadays, most of the applications are dedicated to the investigation of medicinal plants and isolation of natural products (Marston and Hostettmann, 2006; Friesen et al., 2015; Liu et al., 2015).

In this work, we have applied CPC for the fast isolation of the main phenolic constituents from bark of *W. trichosperma.* Additionally, an UHPLC-PDA-MS fingerprint was obtained, and the main compounds were quantified in the medicinal bark. Furthermore, the antioxidant, anti-inflammatory, and enzyme inhibitory effects complemented with the full polyphenolic profile are performed, supporting the reputed medicinal properties of this plant and showing the presence of molecules of pharmacological and industrial interest.

## Materials and Methods

### Chemical and Reagents

Gallic acid (purity > 98%), quercetin, (purity > 97%), 6-hydroxy-2,5,7,8-tetramethylchromane-2-carboxylic acid (Trolox) (purity > 97%), 2,2′-azinobis(3-ethylbenothiazoline-6-sulfonic acid) diammonium salt (ABTS), 2,2-diphenyl-1-picrylhydrazyl (DPPH), Folin-Ciocalteu reagent 2,4,6-tri(2-pyridyl)-s-triazine (TPTZ), aluminum chloride, iron (III) chloride hexahydrate, (4-(2-hydroxyethyl)-1-piperazineethanesulfonic acid (HEPES), ethylenediaminetetraacetic acid (EDTA), adenosine 5′-triphosphate (ATP) disodium salt, acetylcholinesterase from *Electrophorus electricus* (electric eel) and butyrylcholinesterase from equine serum were from Sigma-Aldrich^®^ (Chile). The enzyme 5-lipoxygenase (human, recombinant) were from Cayman Chemical (Item No. 60402). Calcium chloride, sodium carbonate, sodium hydroxide, sodium nitrite, and potassium persulphate were obtained from Merck^®^ (Chile). Acetone, galanthamine, zileuton, sodium acetate trihydrate, and glacial acetic acid were from Merck^®^ (Chile). HPLC reagents, formic acid, methanol, ultrapure water, ethyl acetate, and *n*-hexane were form Merck^®^ (Chile). Deionized water was used for all the experiments.

### Plant Material

The bark of *W. trichosperma* was collected during the months of October and November of 2017 in Valdivia, XIV Region de Los Ríos, Chile, and deposited in our laboratory herbarium (voucher number Wt10012017). The plant was identified by the botanist Alicia Marticorena.

### Extraction of Plant Material

Once the plant material was collected, it was cleaned, dried, and stored at room temperature for 7 days. Then, the bark was cut into pieces and ground to powder in a mill. To obtain the ethanolic extract, or tincture, three macerations were carried out with 540 g of bark powder, 2 L of absolute ethanol, and macerated for 24 h (three times) in darkness at room temperature. Then, the extract was filtered and concentrated in a rotary evaporator to dryness (at 40°C, at 2.7 Bar and 80 r.p.m.), finally, the extract was stored at −80°C in a 50-ml glass bottle. To obtain the edible infusion, 2.5 g of plant material were weighed then 250 ml of boiling deionized water was added, without stirring, to prepare a cup-of-tea infusion of *W. trichosperma*, then it was allowed to stand for 1 h at room temperature and filtered under vacuum, after that the infusion was frozen at −42°C, and finally lyophilized and stored at −80°C in a 1- to 5-ml glass bottle.

### Centrifugal Partition Chromatography

#### Selection of the Solvent System

Centrifugal partition chromatography was applied for the rapid isolation of the main phenolic compounds present in the bark of *W. trichosperma*. The biphasic solvent system used to perform the isolation of the compounds was selected by measuring the partition coefficient (*k*) in different proportions of the hexane-ethyl acetate-methanol-water system (HEMWAT). The value of *k* was defined for the main phenolic compound of the extracts of *W. trichosperma* as *k* = A_upper_/A_lower_, where A_upper_ corresponds to the area under the curve of the peak in the upper phase and A_lower_ corresponds to the area under the curve of the peak in the lower phase of the solvent system, these areas were obtained by gradient elution (**Table 1**), using HPLC-PDA with detection wavelengths at 230, 290, 330, and 380 nm with a flow of 1 ml min^−1^ at 25°C. To obtain the k value, approximately 10 mg of ethanolic extract were weighed in a 2-ml vial, and 1 ml of upper phase and 1 ml of lower phase of the pre-equilibrated biphasic solvent system (HEMWAT 1: 9: 1: 9 v/v, 1: 3: 1: 3 v/v, 2: 5: 2: 5 v/v, and 4: 6: 4: 6 v/v) (Ito, 2005) were added, vortexed, and filtered using a 0.22-μm PTFE filter. Then, 10 ml of each phase was injected in the HPLC-DAD in triplicate to measure the area of each compound. Finally, the biphasic solvent system suitable for the separation of the phenolic compounds of *W. trichosperma* selected.

**Table 1:** Partition coefficient of different solvents systems (HEMWAT).

Solvent system HEMWAT (v/v)	Partition coefficient (*k*) ± SD (*n* = 3)
1:9:1:9	1,550 ± 0,090
1:3:1:3	0,425 ± 0,080
2:5:2:5	0,371 ± 0,102
4:6:4:6	0,054 ± 0,025

#### Separation and Purification of Phenolic Compounds

The biphasic system HEMWAT 1:9:1:9 v/v was used, and 11 g of ethanolic extract were weighed and dissolved in a mixture of 5 ml of upper phase and 5 ml of lower phase, sonicated for 5 min and vortexed for 5 min. The CPC equipment was purged for 1 min with a flow of 30 ml min^−1^, the equipment was filled with stationary phase in normal (or ascending) mode with a rotation of 500 rpm. Once the CPC equipment rotor had a speed of 1800 r.p.m. manual injection of the sample was performed using the injection loop. The mobile phase was supplied using two HPLC pumps with a flow rate of 10 ml min^−1^. The equipment had a PDA detector for the detection of the peaks, the wavelengths used were 280, 330, 350, and 254 nm, the eluent was collected for 1 h by means of an automatic fraction collector for test tubes (25 ml each). Finally, the extrusion was carried out for 15 min with a 100% stationary phase, and the fractions obtained from the majority compounds were lyophilized and subsequently stored in 15-ml glass bottles.

### HPLC Quantitative Analysis

An HPLC-PDA (Thermo Scientific Dionex Ultimate 3000 Series RS, Thermo Fisher Scientific, Germany) was used to determine and quantify the major compounds in the extracts of *W. trichosperma*. A Purosphere Merck^®^ C18-RP column, 25 cm long, particle size 2.5 μm with a precolumn was used, and an elution gradient of water with formic acid (1% v/v) and methanol with formic acid (1% v/v) was used at a flow rate of 1 ml min^−1^, injection volume of 20 μl, and UV detection wavelengths of 230, 290, 330, and 380 nm. Elution gradient of A (water with 1% v/v formic acid) and B (methanol with 1% v/v formic acid). The step gradient was as follows: 0 to 8 min linear increase from 10% to 32% B; then maintaining 32% B, from 8 to 25 min, then, linear increase to 70% B and coming back to initial conditions, then hold 15 min for the next injection. The quantification experiments were performed by HPLC-PDA and elution conditions of qualitative analysis (**Supplementary Material, Figures S1 and S2**). The content of isoastilbin compound and its derivatives were established with reference to calibration curves built with the pure compounds, (astilbin, neoastilbin, and neoisoastilbin) at 254 nm. A stock solution of 2 mg ml^−1^ of purified compounds was used, and the dilutions were prepared to perform calibration curves with the following concentration levels: 1, 5, 10, 30, 50 μg ml^−1^. These dilutions were prepared in triplicate and measured in triplicate (*n* = 9). With the obtained data, the linear regression curve was generated using the Chromeleon 7.2 software, the equation of the curve and the coefficient of determination were obtained (for neoastilbin y = 571.2372x–2.3871, R^2^ = 0.9998; for isoastilbin y = 571.6340x–2.4065, R^2^ = 0.9997, and for neoisoastilbin y = 571.0794x–2.3264, R^2^ = 0.9998). All injections were performed in triplicate, and the results were expressed as mean ± standard deviation (SD).

### MS Conditions

Untargeted analysis of all compounds was performed using a Thermo Scientific Dionex Ultimate 3000 Series RS coupled to an Q-Exactive focus mass spectrometer (Thermo Fisher Scientific, Germany) operated both in positive and negative modes at 17.500 FWHM (*m/z* 200) using a scan range of *m/z* 130–1000 and injection time of 200 ms and automatic gain control at 3e^6^. Scan rate was set at 2 scans s^−1^, and a mass tolerance window was set to 5 ppm. The calibration was performed by positive and negative modes before each series of samples. In addition to the full scan mode, for confirmatory purposes, a targeted MS/MS analysis was performed using the mass inclusion list and expected retention times of the target analytes, with a 30-s time window. Data acquired were finally evaluated by the Browser Xcalibur 2.3 (Thermo Fisher) (Nwokocha et al., 2017).

### Method Performance and Validation of the Analytical Methodology for HPLC-PDA

The validation of the analytical methodology was carried out according to the analytical performance parameters suggested by the ICH (International Council for Harmonization of Technical Requirements for Pharmaceuticals for Human Use) in the guide Q2 (R1) (Borman and Elder, 2018). The accuracy was determined in terms of percent recovery for three points of the calibration curve: 10, 50, and 500 μg ml^−1^. The accuracy was assured for the limit of quantification. In addition, the percentage of absolute recovery was determined.

Extraction with boiling water was carried out with 50.0 mg of powdered plant material, for that 0.3 mg (100%), 0.4 mg (130%), and 0.7 mg (150%) of isoastilbin were added. The repeatability (intraday precision) was determined in triplicate of six points of the calibration curve by the same analyst in the same working day. The accuracy under the repeatability conditions was expressed in relative standard deviation (RSD). The intermediate precision was determined by the same analyst in different days. Three points of the calibration curve were prepared, meaning low, medium, and high concentration in triplicate. The intermediate precision was expressed in RSD. The range of work chosen was between 0.01 (lower limit) and 0.5 mg ml^−1^ (upper limit) of isoastilbin and as intermediate concentrations 0.03, 0.05, 0.1, and 0.3 mg ml^−1^. The limit of detection (LOD) was determined based on the signal-to-noise ratio (3:1), and the limit of quantification (LOQ) was determined based on the signal-to-noise ratio (10:1). The method was validated; as seen in **Supplementary Material, Tables S1–S6**. According to the results all values were within acceptable criteria (Borman and Elder, 2018). The LOD and LOQ values were lower than 0.12 and 0.39 mg standard respectively (**Supplementary Material, Table S1**). Therefore, the method was concluded as suitable to quantify isoastilbin and related phenolic compounds in the plant extract. Over the selected range, peak areas linearly depended on concentrations for all phenolic compounds with high correlation coefficients (>0.9998, **Table 1**). The injection repeatability of the system was evaluated by seven consecutive injections of the standard solution of phenolic compounds. The RSD values of retention times of phenolic compounds were less than 0.60% (**Supplementary Material, Table S6**).

### Determination of Total Phenolics and Flavonoids Content

The spectrophotometric quantification of total phenolic content (TPC) and total flavonoid content (TFC) of the lyophilized aqueous extract (infusion) and tincture of the bark of *W. trichosperma* was carried out following the previously reported Folin-Ciocalteu and AlCl_3_ methodology in a Synergy HTX microplate reader (Biotek, Winoosky, VT, USA) (Kuskoski et al., 2005; Bhaigyabati et al., 2017). Total phenolics using the Folin-Ciocalteu reagent, the results were derived from a calibration curve of gallic acid (y = 0.0319x + 0.138, R^2^ = 0.996). Also, for total flavonoids, the results were derived from the calibration curve of quercetin (y = 0.0136 x + 0.0414, R^2^ = 0.979). The bark was tested at 1 mg ml^−1^, Total phenolic content was expressed as milligrams of gallic acid equivalents (GAE) per gram of extracts (mg GAE g^−1^ extract). Flavonoids were expressed as milligrams of quercetin equivalents (QE) per gram of extracts (mg QE g^−1^ extract). The determinations were made in triplicate. The values from triplicates are reported as the mean ± SD.

### *In Vitro* Antioxidant Activity of *W. trichosperma*

The antioxidant activity was evidenced by *in vitro* assays such as the oxygen radical absorbance capacity (ORAC), bleaching of the free radical 2,2-diphenyl-1-picrylhydracil hydrate (DPPH), Ferric Reducing Antioxidant Power (FRAP) and by the bleaching of 2,2′-azino-bis-(3-ethylbenzothiazoline-6-sulphonic acid (ABTS). These assays were performed for the extracts and the major compound isolated, isoastilbin. Data were recorded in triplicate and expressed as average value.

### ABTS Assay

The ABTS free radical scavenging assay was employed to measure the free radical scavenging activity of extracts of *W. trichosperma* and for isoastilbin. We used the method of Re et al. (1999) with some modifications, a solution of 7 mM ABTS (2,2′-azino-bis(3-ethylbenzothiazoline-6-sulphonic acid)) and a solution 2.45 mM potassium persulfate in water was prepared. Both aqueous solutions were mixed in a 1:1 ratio (v/v) and incubated for 16 h in the dark for the formation of the radical at room temperature. After this period, using 96-well plates a volume of 275 µl of solution (absorbance 0.7) was mixed thoroughly with 25 µl of standard or the samples, the absorbance was immediately recorded at 734 nm using a microplate reader. Gallic acid was used as a reference standard (from 1 to 100 µg ml^−1^); a curve of extracts of *W. trichosperma* were made (from 19.53 to 625 µg ml^−1^) and a curve of isoastilbin (from 19.53 to 2500 µg ml^−1^) to calculate their IC_50_. The percentage of ABTS radical inhibition was calculated according to the following equation:

$$\mathrm{ABTS}\;\mathrm{inhibition}\;\left(\%\right)=\left(1-\frac{\mathrm{As}-\mathrm{Abs}}{A:\mathrm{ABTS}}\right)\times 100$$

As, is the absorbance of the sample with ABTS; Abs, is the absorbance of the mixture of blank and sample; A: ABTS, is the absorbance of the ABTS stock solution. The IC_50_ was calculated using GraphPad Prism 7 software.

### DPPH-Free Radical-Scavenging Activity Assay

DPPH free radical-scavenging activity was determined according to Brand-Williams et al. (1995) with some modifications. A solution of DPPH 400 µM was prepared and using a volume of 150 µl of this solution thoroughly mixed with 50 µl of standard or the samples, the absorbance was recorded at 515 nm using a microplate reader and 96-well plates 30 min later. Gallic acid was used as a reference standard (from 1 to 100 µg ml^−1^); curves of extracts of *W. trichosperma* (from 19.53 to 625 µg ml^−1^) and a curve of isoastilbin (from 19.53 to 2500 µg ml^−1^) were prepared. The percentage of DPPH radical inhibition was calculated according to the following equation:

$$\mathrm{DPPH}\;\mathrm{inhibition}\;\left(\%\right)=\left(1-\frac{\mathrm{As}-\mathrm{Abs}}{\mathrm{A DPPH}}\right)\times 100$$

As is the absorbance of the sample with DPPH, Abs is the absorbance of the mixture of blank and sample, A DPPH is the absorbance of the DPPH stock solution. The IC_50_ was calculated using GraphPad Prism 7 software.

### Ferric Reducing/Antioxidant Power FRAP Assay

The method of Collins et al. (1959) with some modifications was used, briefly, a buffer solution CH_3_Na*3H_2_O 3.1%/CH_3_COOH (glacial) 16%, dissolved in water plus 20 mM FeCl_3_ in aqueous solution HCl 0.02 M and 10 mM TPTZ dissolved in absolute ethanol. The working solution corresponded to a mixture of one volume of buffer with one volume of FeCl_3_ and 11 volumes of ethanol. Trolox was used as a reference standard (from 10 to 250 µg ml^−1^), for each level of concentration, 290 µl of working solution were mixed with 10 µl of Trolox in a well of the microplate in triplicate, and then the absorbance was measured at 593 nm after 5 min, with the data obtained a linear regression equation was used to calculate the concentration of samples.

### ORAC Assay

The assay was carried out on a plate reader, at 37°C as reported (Wu et al., 2004). Briefly, AAPH was used as peroxyl generator and Trolox as a standard. Using 48-well microplates, 40 µl of sample, blank, and Trolox calibration solutions were mixed in triplicate. The fluorescence of fluorescein disodium (FL) of each cycle was recorded. Parameters for the plate reader were: orbital shaking (4 mm shake width), cycle time, 210 s; position delay, 0.3 s; cycle number, 35; shaking mode, 8 s before each cycle; injection speed, 420 µl s^−1^. Values were calculated by using a quadratic regression equation between the Trolox or sample concentration and net area under the FL decay curve. Data are expressed as micromoles of Trolox equivalents (TE) per gram of sample (µmol of TE g^−1^). The area under curve (AUC) was calculated as

AUC=0.5+f4/f3+f5/f3+f6/f3+⋯+f32/f4+f33/f4)×CT

where f3 = fluorescence reading at cycle 3; f*n*= fluorescence reading at cycle *n*; and CT, cycle time in minutes.

### Enzyme Inhibitory Activity

#### Cholinesterases (ChE) Inhibition

The inhibitory activity of these important enzymes was measured thorough the Ellman’s method, as stated previously (Mocan et al., 2017). Briefly, DTNB was dissolved in buffer Tris-HCl at pH 8.0 containing 0.1 M NaCl and 0.02 M MgCl_2_. Then, a filtered sample solution dissolved in deionized water (50 ml, 2 mg ml^−1^) was mixed with 125 ml of 5-dithio-bis(2-nitrobenzoic) acid (DTNB), acetylcholinesterase (AChE), or butyrylcholinesterase (BChE) solution (25 ml) dissolved in Tris-HCl buffer at pH 8.0 in a 96-well microplate and incubated for 15 min at 25^°^C. Initiation of reaction was performed by the addition of acetyl-thiocholine iodide (ATCI) or butyryl-thiocholine chloride (BTCl) (25 ml). In addition, a blank was prepared by adding the solution sample to all reagents without the enzyme(s) (AChE or BChE) solutions. The sample and blank absorbances were then read at 405 nm after 10 min of incubation at 25°C. The absorbance of the sample was subtracted from that of the blank and the cholinesterase inhibitory capacity was expressed as IC_50_ (µg ml^−1^, concentration range 0.05 to 25 µg ml^−1^). Galantamine was used as positive control. All data were collected in triplicate.

#### Human 5-lipoxygenase (5-hLOX) Inhibition

The screening inhibitory activity of 5-hLOX was performed fluorescence-based assay is performed using microplates. For this, a 50-mM HEPES buffer was prepared at pH 7.5, which contains 2 mM EDTA, 2 mM CaCl_2_, and 10 μM ATP. A dilution of the human 5-lipoxygenase (5-hLOX) enzyme (1:500) in the reaction buffer was used together with 10μM 2,7-dichlorofluorescein diacetate (H2DCFDA dye, fluorimetric probe). The mixture was allowed to incubate in the assay microplate for 15 min at room temperature. Subsequently, 33.3 μl of the buffer was added to each well of the cell culture plate to be used. To each well, 3.3 μl of inhibitor was added and incubated for 30 min. Finally, the reaction was started by the addition of a suitable concentration of arachidonic acid substrate (3.3 µl, 0.5 μM), and fluorescence was read in a Synergy™ HT Multi-Mode Microplate Reader (Biotek Instruments, Inc, Winooksi, VT, USA) at 480 nm excitation/520 nm emission after a reaction that had proceeded for 1 h at room temperature for determine the values of percentage inhibition. Nordihydroguaiaretic acid (NDGA) was used as a positive control. All data were collected in triplicate.

### Docking Studies

The geometries and partial charges of astilbin, isoastilbin, neoastilbin, and neoisoastilbin contained in the *W. trichosperma* aqueous extract, as well as the known cholinesterases (*Tc*AChE- *h*BuChE) inhibitor galantamine and the 5-*h*LOX inhibitor zileuton were fully optimized using the DFT method with the standard basis set PBE0/6-311+g* (Adamo and Barone, 1999, Petersson et al., 1988). All calculations were performed in Gaussian 09W software.

### Statistical Analysis

Results were expressed by means of values ± standard error of nine separate determinations (GraphPad Prism 8). Comparison of means was performed by one-way analysis of variance (ANOVA) (p < 0.05) followed by *post hoc* Tukey honestly significant difference (HSD) test (p < 0.01).

## Results and Discussion

### Centrifugal Partition Chromatography

The major phenolic compounds present in the extracts, isoastilbin, neoisoastilbin, and neoastilbin ((2*R*,3*S*)-dihydroquercetin 3-*O*-alpha-l-rhamnoside, (2*S*,3*R*)-dihydroquercetin 3-*O*-alpha-l-rhamnoside, (2*S*,3*S*)-dihydroquercetin 3-*O*-alpha-l-rhamnoside) were isolated using a countercurrent chromatography (CCC) machine, and a biphasic solvent system HEMWAT 1:9:1:9. The partition coefficient is the parameter that reflects the distribution of the solute between the two mutually balanced solvent phases. The ideal partition coefficient, for optimum CCC performance, must be a *k* value between 0.4 and 2.5 (Friesen and Pauli, 2005; Kumar, 2015). It is necessary to point out that to obtain an efficient separation and an adequate execution time the value k should be close to 1.0 (Kumar, 2015). On the one hand, in the event that the partition coefficient is much smaller than 1.0, the loss of the maximum resolution results as the solutes would be close to each other on the solvent front (Kumar, 2015). On the other hand, in the case that the value of *k* is much greater than 1, elution of the solutes in excessively wide peaks (band widening) would occur, which can lead to a prolonged elution time (Kumar, 2015). For all the above, the biphasic solvent system 1:9:1:9 (v/v) with a *k* value equal to 1.55 ± 009 was chosen (**Table 1**). This solvent system allowed to separate in a step the main compound present in the ethanolic extract of *W. trichosperma*.

### HPLC-PDA-OT-MS

**Figure 2:**
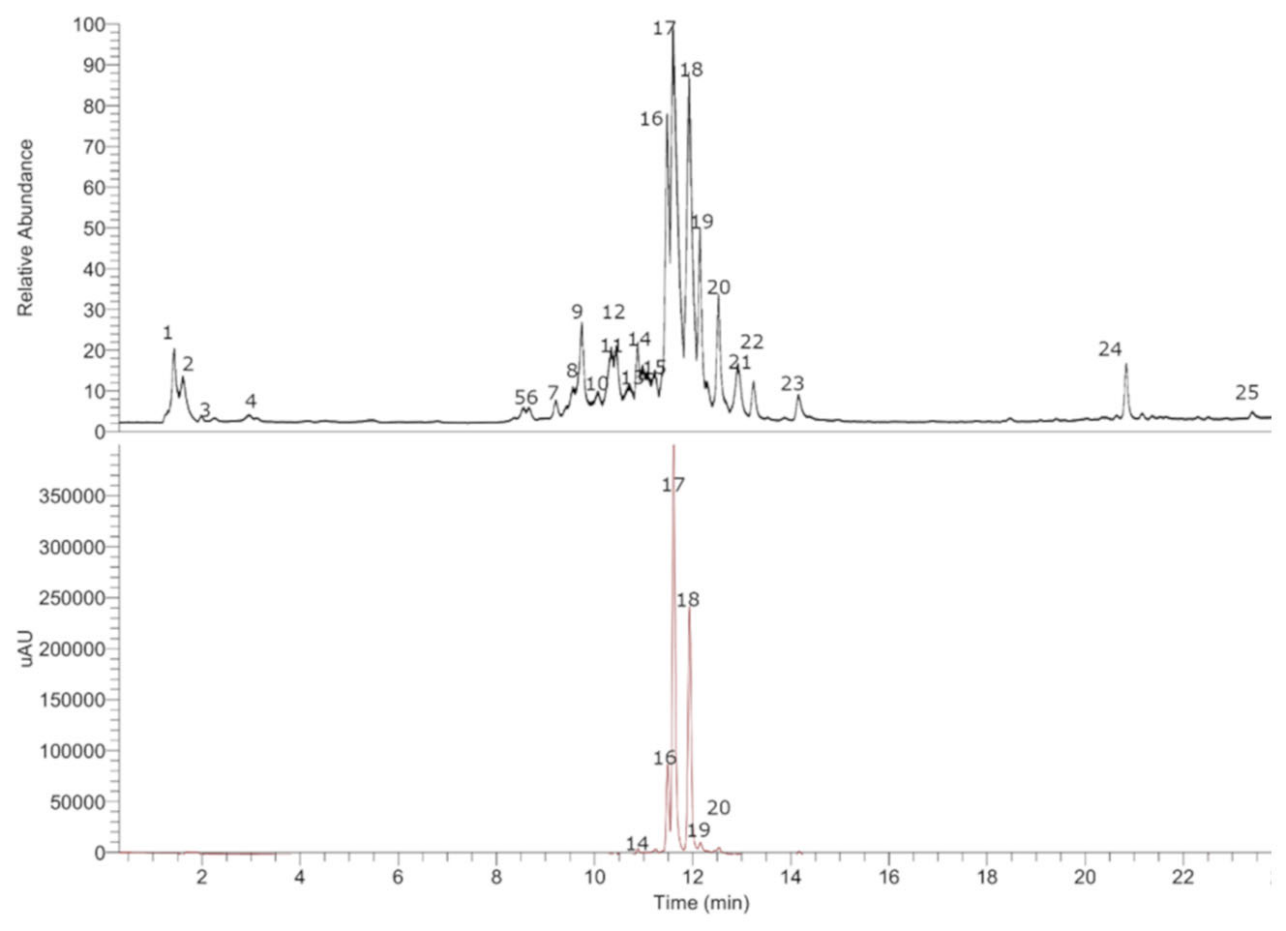
HPLC-ESI-MS/MS profile (black: TIC, red: UV at 280 nm) of the infusion of bark from *W. trichosperma*.

In this study, through HPLC-PDA-OT-MS analysis, twenty-five compounds were tentatively identified in the ethanolic extract of *W. trichosperma* bark (**Figure 2**). The structures of the compounds were proposed based on UV absorption and MS fragmentation patterns (**Table 2**). The detailed analysis is explained below.

**Table 2:** Tentative identification of main compounds of *W. trichosperma* by HPLC-PDA-OT-MS.

Peak	R_t_ (min)	λ max (nm)	Elemental composition [M-H]^-^	Theorical mass (*m/z*)	Measured mass (*m/z*)	Accuracy (δ ppm)	Ions MS^n^ (m/z)	Tentative identification
1	1.42		C_12_H_7_O_4_^-^	215.03222	215.03389	−7.8		Bergapten
2	1.62		C_15_H_13_O_6_^-^	331.06720	331.06597	3.71	125.02363	Galloylglucose
3	2.24		C_15_H_13_O_6_^-^	331.06689	331.06595	3.70	125.02361	Galloylgalactose
4	2.96	198-230-293	C_15_H_13_O_6_^-^	169.01369	169.01315	4.58	125.02364	Gallic acid
5	8.23	198-230-293	C_19_H_27_O_13_^-^	463.14523	463.14462	4.58	285.04062 151.00308	Phlorigidoside B
6	8.65	198-230-293	C_20_H_29_O_12_^-^	461.16614	461.16535	4.58	285.06122 (catechol)	Verbasoside
7	9.21	198-230-293	C_30_H_25_O_12_^-^	577.13403	577.13405	0.35	285.06122 (catechol)	Procyanidin B2
8	9.53	198-230-293	C_17_H_22_O_12_^-^	417.10358	417.10275	1.97		Tudoside
9	9.75	198-230-293	C_15_H_13_O_6_^-^	289.07066	289.07199	4.58		Epicatechin
10	10.42	198-230-293	C_21_H_23_O_10_^-^	435.12857	435.12936	0.76		Phlorizin
11	10.22	198-230-293	C_21_H_23_O_13_^-^	483.11332	483.11450	2.64	285.04062 151.00308	Hedanthroside E
12	10.46	283	C_21_H_21_O_12_^-^	465.10345	465.10275	1.50	269.04437, 161.02782	Taxifolin-3-*O*-glucoside
13	10.55	283	C_21_H_21_O_12_^-^	465.10358	465.10275	1.77	281.10859, 339.07106, 161.02690	Helicioside B Taxifolin-5-*O*-glucoside
14	11.22	198-230-293	C_15_H_15_O_9_^-^	339.07216	339.07239	4.10	176.12755	Esculin
15	11.35	280	C_20_H_19_O_11_^-^	435.09308	435.09219	2.04	C_15_H_9_O_6_^-^ (naringenin)	Naringenin-3-*O*-pentoside
16	11.46	198-230-293	C_21_H_21_O_11_^-^	449.10894	449.10922	3.08	285.04062 151.00308	Neoastilbin
17	11.61	198-230-293	C_21_H_21_O_11_^-^	449.10784	449.10907	2.74	285.04062 151.00308	Isoastilbin
18	12.02	198-227-290	C_21_H_21_O_11_^-^	449.10784	449.10870	−3.09	–	Neoisoastilbin
19	12.23	198-230-293	C_21_H_21_O_11_^-^	449.10784	449.10901	2.60	285.04062 151.00308	Astilbin
20	12.16	203	C_28_H_25_O_15_^-^	433.11560	433.11429	−3.15	269.04995, 285.04055 152.01096	Naringenin-4´*-O*-glucoside
21	12.52	286	C_28_H_25_O_15_^-^	601.11880	601.11651	1.18	285.03967	Eriodictyol 7-(6`-galloylglucoside)
22	12.92	288	C_28_H_25_O_15_^-^	433.11560	433.11374	−3.15	271.06120 152.01096	Naringenin-7*-O*-galactoside
23	14.16	266-293	C_15_H_10_O_6_^-^	285.04028	285.03936	3.22	–	Kaempferol
24	20.84	283	C_12_H_15_O_2_^-^	191.10732	191.10666	3.44	–	Pentyl benzoate
25	23.40	283	C_15_H_22_O_2_^-^	233.15436	233.15361	3.35	–	Polygodial

#### Phenolic Acids

Peak 1 was tentatively identified as bergapten (215.03389, C_12_H_7_O_4_^-^), (Acharya et al., 2019) peaks 2 and 3 as the isomers galloylglucose (331.06597, C_15_H_13_O_6_^-^) (Salminen et al., 2014), and galloylgalactose (C_15_H_13_O_6_^-^), respectively. Peak 4 with a M-H^-^ ion at *m/z*: 169.01315 as gallic acid (C_15_H_13_O_6_^-^), Peak 5 as phlorigidoside B (463.14462 C_19_H_27_O_13_^-^) (Takeda et al., 2001), and finally peak 6 as verbasoside (461.16535 C_20_H_29_O_12_^-^) (Quirantes-Piné et al., 2009), while peak 24 was identified as pentyl benzoate, (435.09219, C_20_H_19_O_11_^-^).

#### Procyanidins

Peak 7 with a pseudomolecular ion at *m/z*: 577.13405 producing a catechol moiety at *m/z*: 285.06122 was identified as procyanidin B2 (C_30_H_25_O_12_^−^) (Glavnik and Vovk, 2019), while peak 9 as epicatequin (289.07199).

#### Iridoids

Peak 8 was identified as tudoside (417.10275, C_17_H_22_O_12_^-^)(Venditti et al., 2015), peak 11 as hedanthroside E. (483.11450, C_21_H_23_O_13_^-^)

#### Chalcones

Peak 10 with a M-H ion at *m/z*: 435.12936 (C_21_H_23_O_10_^-^) was identified as phlorizine (Liu et al., 2003)

#### Flavonoids

Peak 12 was identified as the flavanonol taxifolin-3-*O*-glucoside (C_21_H_21_O_12_^−^) (Sakushima et al., 2002) and peak 13 as its isomer helicioside A (Jia et al., 2018) and peak 15 as naringenin-7-*O*-pentoside (C_20_H_19_O_11_^-^). Peaks 20, 21, and 22 as naringenin-4′-*O*-glucoside, eriodictyol 7-(6´´-galloylglucoside) (C_28_H_25_O_15_^-^) and naringenin-7-*O*-glucoside (prunin, C_28_H_25_O_15_^-^) (Ali et al., 2019), respectively. Finally, Peak 24 was identified as kaempferol (C_15_H_10_O_6_^-^).

#### Flavanones

**Figure 3:**
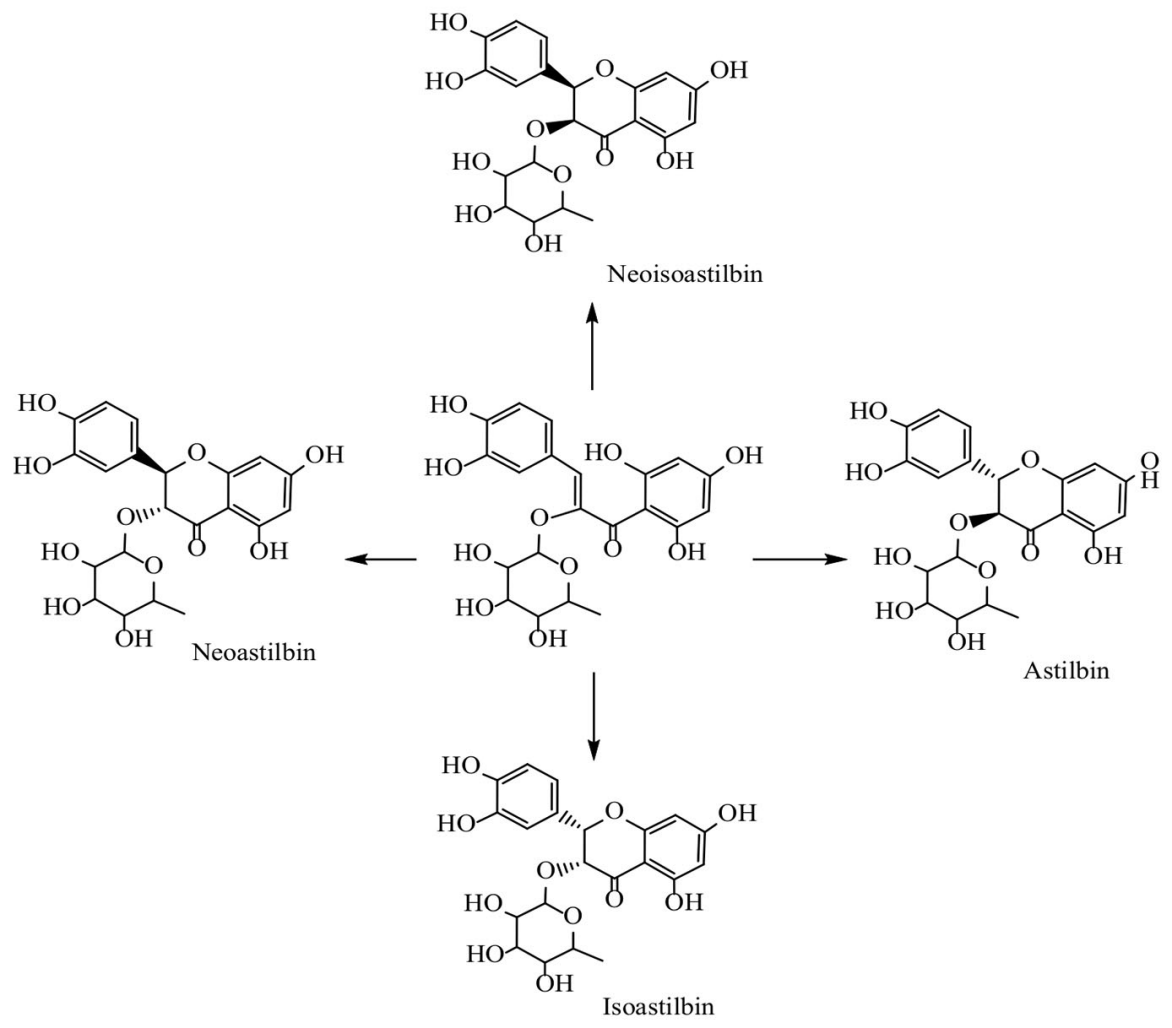
Biosynthetic relationship of the isomers of isoastilbin detected in the bark of *W. trichosperma*.

Peaks 16 to 19 were identified as neoastilbin, isoastilbin, neoisoastilbin, and astilbin (**Figure 3**). (C_21_H_21_O_11_^-^) (Zhang et al., 2013), respectively, using spiking experiments with standard compounds. The structural identification of isoastilbin was confirmed by NMR analysis (**Supplementary Material, Figures S3–S9**).

#### Coumarins

Peak 14 was identified as esculin (339.07239, C_15_H_15_O_9_^-^) (Qazi et al., 2018)

#### Sesquiterpenes

Peak 25 was regarded as polygodial (Barrosa et al., 2016).

### HPLC-PDA Quantitation

Isomers of astilbin were quantified by HPLC-PDA and the methodology validated; (as seen in **Supplementary Material, Tables S1–S6**), the method was suitable to quantify isoastilbin and related phenolic compounds in the bark extract. In the infusion, four isomers were found but only three mayor isolated ones quantified, the highest amount found was for isoastilbin, followed by neoisoastilbin, and neoastilbin (141.7366 ± 1.18, 99.59 ± 0.83, and 53.1795 ± 0.31 mg/g extract, respectively), this makes the bark a good source for those bioactive isomer glycoside compounds.

### Total Phenolic Content, Total Flavonoid Content, and Antioxidant Activity

Total phenolic content (TPC) and total flavonoid content (TFC) was measured of the aqueous extract of *W. trichosperma* to determine if the antioxidant activity was directly related to the content of these metabolites. The content of phenolic compounds in aqueous extracts of *W. trichosperma* was 442.1 ± 21.1 mg GAE g^−1^ dry plant (**Table 3**). The flavonoid content in aqueous extract was 1.554 ± 0.111 μg of quercetin equivalents per gram of dry plant. On the other hand, we used the ABTS^•+^ and DPPH assays to perform the assessment of the bleaching capacities of the aqueous extract of *W. trichosperma* and the major compound isolated, isoastilbin, the two assays showed a concentration-dependent increases in radical scavenging activity both the extract and isoastilbin, and we compared the data with the IC_50_ value of standard gallic acid for each method, as presented in **Table 3**, for ABTS assay the IC_50_ value for gallic acid was 0.09855 ± 0.00026 μg of gallic acid per milliliter and for DPPH assay was 0.4622 ± 0.0015 μg of gallic acid per milliliter. Also, for FRAP assay, the results were derived from a calibration curve of Trolox (y = 0.0019x + 0.0178, R^2^ = 0.997), for the aqueous extract this was 2.700 ± 0.554 mmol Trolox equivalent per gram of dry plant and for isoastilbin this was 0.1494 ± 0.0166 mmol Trolox equivalent per milligram of isoastilbin. For ORAC assay, the results support the hypothesis that the antioxidant activity is related with the content of the phenolic compounds present in the extract of the bark of *W. trichosperma*.

**Table 3:** Total phenolics, flavonoids, antioxidant, and inhibition of cholinesterase and 5-hLOX for infusion and tincture of *W. trichosperma* and the main isolated compound, isoastilbin.

Assay	Infusion of *W. trichosperma*	Tincture of *W. trichosperma*	Gallic acid	Isoastilbin	Galantamine/NDGA
**TPC**	442.1 ± 21.1^a^	438.3 ± 22.2^a^	–	–	–
**TFC**	15.54 ± 0.11^b^	15.45 ± 0.11^b^	–	–	–
**DPPH IC_50_**	20.58 ± 2.46^c^	20.33 ± 2.18^c^	0.4622 ± 0.0015^k^	33.1 ± 2.1^d^	–
**ABTS IC_50_**	3.070 ± 0.095^c^	3.055 ± 0.082^c^	0.0985 ± 0.0003^k^	2.525 ± 0.261^d^	–
**FRAP**	2.700 ± 0.554^e^	2.650 ± 0.532^e^	–	0.149 ± 0.016^f^	–
**ORAC**	1270 ± 36.5^g^	1248 ± 32.3^g^	–	1901± 33.5^g^	–
**Acetylcholinesterase inhibition IC_50_**	3.13 ± 0.03^h^ (33.80% at 1 µg ml^−1^)	3.08 ± 0.02^h^ (33.50% at 1 µg ml^−1^)	–	4.68 ± 0.03^h^ (51.70% at 2.2 µM)	0.26 ± 0.02^h^ (69.42% at 3.48 µM)
**Butirylcholinesterase inhibition IC_50_**	2.94 ± 0.08^h^ (33.12% at 1 µg ml^−1^)	2.76 ± 0.08^h^ (33.12% at 1 µg ml^−1^)	–	8.51 ± 0.03^h^ (50.10% at 2.2 µM)	3.82 ± 0.02^h^
**5-hLOX inhibition** **(%)**	82.86 ± 4.31 (at 0.05 µg ml^−1^)^i^	–	–	34.29 ± 3.11 (at 10 µM)^i^ 80.71 ± 7.29 (at 40 µM)^i^	93.50 ± 4.2 (at 10 µM)^i^

All values were expressed as means ± SD (n = 3).

^a^expressed in mg of gallic acid equivalents per gram of dry plant. ^b^expressed in mg of quercetin equivalents per gram of dry plant.^c^expressed in μg of extract per ml. ^d^expressed in μg of isoastilbin per milliliter. ^e^expressed in mmol Trolox equivalent per gram of dry plant. ^f^expressed in mmol trolox equivalent per mg of isoastilbin. ^k^expressed in μg of gallic acid per ml. ^g^expressed in µM Trolox equivalents per 100 grams of dry plant. ^h^IC_50_ in µg per ml. ^i^Concentration Stock Solution equal to 1.52 mg per ml^−1^.

### *In Vitro* 5-hLOX and Cholinesterase Inhibitory Enzyme Assay

The application of enzyme inhibition therapeutic approaches is considered effective to control several health degenerative diseases, including Alzheimer’s disease, arthritis, and arthrosis. LOXs are a class of enzymes that catalyze the oxygenation of poly-unsaturated fatty acids in *cis*,*cis*-1,4-pentadiene structures, which are classified in order to their arachidonic acid oxidation specificity as 5, 8, 12, and 15-LOX. Indeed, the enzymes cyclooxygenase (COX), lipoxygenase (LOX), and matrix Metalloproteinase (MMP) are closely related to inflammatory responses, key factors in joint and arterial health. They are important key factors in immunity responses and pathological processes, such as wound healing, arthrosis, rheumatoid arthritis, pain, cancer, fever, and inflammatory reactions. On the other hand, the first therapeutic line for the treatment of degenerative Alzheimer’s disease are cholinesterase inhibitors (Howes et al., 2003). In this study, an *in vitro* inhibitory effect of the aqueous extract of *W. trichosperma*, on 5-hLOX, acetylcholinesterase, and butyrilcholinesterase enzymes was investigated.

The aqueous extract of *W. trichosperma* showed an interesting activity as 5-hLOX inhibitor of 82.86% (at 0.05 μg ml^−1^), while isolated isoastilbin was 34.29% (at 10 µM) and 80.71% (at 40 µM) (**Table 3**). Within the identified compounds, neoastilbin, isoastilbin, neoisoastilbin, and astilbin have shown anti-inflammatory activity through a mechanism that related to lipopolysaccharide-stimulated PGE(2) release, and Tumour Necrosis Factor alpha (TNF-α) and nitric oxide (NO) production from RAW 264.7 cells (Qureshi et al., 2011). In addition, Bergapten exhibited significant inhibition of the production of pro-inflammatory cytokines, namely, TNF-α and interleukin-6 (IL-6) by human peripheral blood mononuclear cells (PBMCs) stimulated with lipopolysaccharide in a concentration-dependent manner (Bose et al., 2011), and also exhibit an anti-inflammatory activity by inhibiting reactive oxygen species (ROS) and NO accumulation in the tail-cutting-induced inflammation zebrafish model (Yang et al., 2018b). Gallic acid suppresses the activation of nuclear factor kappa-light-chain-enhancer of activated B cells (NF-*k*B), COX-1, COX-2, p65 acetylation, Histone acetyltransferases (HAT), Intercellular adhesion molecule-1 (ICAM-1), Vascular cell adhesion molecule 1 (VCAM-1), TNF-α, interleukin-1β (IL-1β), IL-6, and inducible nitric oxide synthase (iNOS) (Kroes et al., 1992; Locatelli et al., 2013). Procyanidin B2 exerts anti-inflammatory activity and molecular mechanisms targeted include the modulation of various mediators of inflammation, including eicosanoids, cytokines, and NO, NLR Family Pyrin Domain Containing 3 (NLRP3) inflammasome, as well as the NF-κB and mitogen-activated protein kinase (MAPK) pathways (Martinez-Micaelo et al., 2012; Jiang et al., 2018). Epicatechin effectively attenuates the production of inflammatory mediators including NO, PGE2, TNF-α, IL-1β, and IL-6 in the LPS-induced macrophages through inactivation of NF-κB, MAPKs (ERK, JNK, and p38) and JAK2/STAT3 pathways (Yang et al., 2015). Esculin has potent anti-inflammatory activities *in vivo* and *in vitro*, which may involve the inhibition of the MAPK pathway (Niu et al., 2015). Kaempferol showed potent effect in the inhibition of inflammatory cell function as well as inhibition in the expression of pro-inflammatory cytokines and chemokines (Devi et al., 2015). Polygodial inhibited significantly the mouse paw edema induced by prostaglandin E2, bradykinin (BK), substance P (SP), dextran, platelet activating factor (PAF) or carrageenan, and also inhibited arachidonic acid-, capsaicin- and croton oil-induced ear edema in mice (da Cunha et al., 2001). Flavanones were regarded previously as anti-inflammatory showing inhibitory effects on lipopolysaccharide-stimulated PGE(2) release, and TNF-α and NO production from RAW 264.7 cells (Qureshi et al., 2011).

The inhibition of both the AChE and BChE cholinesterase enzymes is believed to be beneficial over inhibiting only one of the enzymes. The results of *W. trichosperma* inhibitory potencies of choline esterase (AChE and BChE) inhibitory activities are shown in **Table 3** and expressed as IC_50_, particularly, AChE inhibition IC_50_ was 3.13 ± 0.03 µg ml^−1^ (33.8% at 1 µg ml^−1^) and BChE inhibition IC_50_ was 2.94 ± 0.08 µg ml^−1^ (33.12% at 1 µg ml^−1^) while its component isomer isoastilbin its IC_50_ was 4.68 ± 0.03 µg ml^−1^ (51.70% at 2.2 µM) and 8.51 ± 0.03 µg ml^−1^ (50.10% at 2.2 µM) for AChE and BChE, respectively.

### Docking Studies

**Figure 4:**
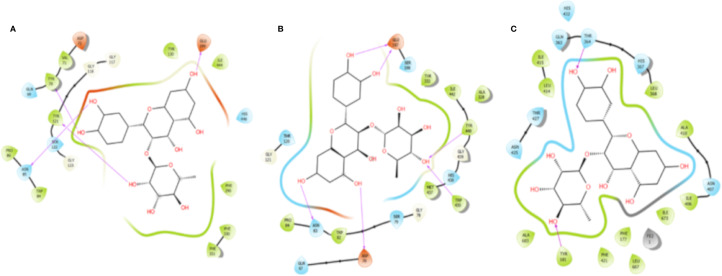
Hydrogen bond interactions showed by isoastilbin into the catalytic site of the three studied enzymes. **(A)** Isoastilbin into acetylcholinesterase (*Tc*AChE) catalytic site **(B)** Isoastilbin into butyrylcholinesterase (*h*BuChE) catalytic site. **(C)** Isoastilbin into 5-lipoxygenase (5- *h*LOX) catalytic site. Magenta arrows represent H-bondings with the different amino acids.

The isomers astilbin, isoastilbin, neoastilbin, and neoisoastilbin (**Supplementary Material, Figure S10**) contained in *W. trichosperma* as well as the known inhibitors of acetylcholinesterase (*Tc*AChE), butyrylcholinesterase (*h*BuChE) and 5-lipoxygenase (5-*h*LOX), galantamine, and zileuton, were subjected to docking assays into their catalytic sites. The best docking binding energies expressed in kcal/mol of each compound are shown in **Table 4**. By the analysis of docking poses in this enzyme, astilbin and isoastilbin are arranged in similar manners with a slightly tilt between their molecular structures. Indeed, the 5,7-dihydroxychroman-4-one, dihydroxyphenyl, and glycoside portions are quite overlapped, which could explain the similarities in their binding energy values (−8.92 and −8.59 kcal mol^−1^, respectively). The relative superposition between astilbin and isoastilbin mentioned above results in that both isomers interacts with some same amino acids of the acetylcholinesterase catalytic site (**Supplementary Material, Figures S11A, B**). In this sense, the hydroxyl groups (–OH) at position 7- of the 5,7-dihydroxychroman-4-one frameworks, one of the hydroxyl groups (–OH) of the glycosides moieties and one of the hydroxyl groups (–OH) of the dihydroxyphenyl cores of both astilbin and isoastilbin molecules showed hydrogen bond interactions with the amino acids Glu199, Tyr121, and Asn85, respectively. The only difference between the aforementioned interactions of these two molecules is specifically in the Hydrogen bond interaction performed by the hydroxyl group (–OH) of the glycoside core in astilbin, which take place between the oxygen atom of the saccharide and the hydrogen atom of Tyr121, whereas in isoastilbin occurs exactly the opposite. **Figure 4** shows the Hydrogen bond interactions in a two-dimensional diagram of isoastilbin binding mode into every catalytic site of the three studied enzymes as a pure and sole inhibitor in experimental inhibition assays. Binding energy data of flavonoids astilbin, isoastilbin, and neoastilbin obtained from docking assays over acetylcholinesterase shown in **Table 4**, exhibited similar values. This suggest that all these isomers possess similar ability to inhibit this enzyme. The similar binding energy values of these three compounds could explain why the experimental IC_50_ of the *W. trichosperma* aqueous extract and the IC_50_ of the isolated isoastilbin showed such close values (4.68 ± 0.03 and 3.13 ± 0.32 µg ml^−1^, respectively). On the other hand, neoisoastilbin presented the best binding energy profile, which could lead to a higher inhibitory potency if this compound were isolated and tested as a single inhibitor. Therefore, since the *W. trichosperma* aqueous extract contain the four isomers (astilbin, isoastilbin, neoastilbin, and neoisoastilbin) the eventual higher potency of neoisoastilbin compared to the other three isomers, it cannot be detected due the existing competition among all flavonoids for the acetylcholinesterase catalytic site.

**Table 4:** Binding energies obtained from docking experiments of astilbin, isoastilbin, neoastilbin, neoisoastilbin, and the respective known enzyme inhibitors galantamine and zileuton over acetylcholinesterase (*Tc*AChE) (**Supplementary Material, Figure S11**), butyrylcholinesterase (*h*BuChE) (**Supplementary Material, Figure S12**) and 5-lipoxygenase (5- *h*LOX) (**Supplementary Material, Figure S13**).

Compound	Binding energy (kcal/mol) Acetylcholinesterase (*Tc*AChE)	Binding energy (kcal/mol) Butyrylcholinesterase (*h*BuChE)	Binding energy (kcal/mol) 5-lipoxygenase **(5- *h*LOX)**
Astilbin	−8.92	−9.41	−3.61
Isoastilbin	−8.59	−9.25	−1.17
Neoastilbin	−9.78	−9.38	−1.3
Neoisoastilbin	−10.37	−8.98	2.33
Galantamine	−11.81	−9.5	-
Zileuton	-	-	−7.25

## Conclusions

This study deals with the antioxidant, and enzyme inhibitory effects of *Weinmannia trichosperma*, in order to find new endemic and underutilized sources of natural products for potential applications in human health. This study makes new contributions to the chemistry of this plant, not previously described. The CPC machine was useful for the isolation of the major compounds while the HPLC-MS allowed the identification of several phenolic constituents. In this study, the assessment of antioxidant activity indicates that the bark could be an important source of natural antioxidants and inhibitory compounds for potential use in neurodegenerative diseases. Hence, *W. trichosperma* bark could be recommended for the formulation of nutraceuticals or antioxidant-rich therapeutic diets and for its enzyme inhibitory properties it can have potential applications in drug industry for the develop of treatment for Alzheimer’s disease, as well as to alleviate inflammatory processes involved in other pathologies and diseases. However, more detailed *in vivo* studies are needed to evaluate the safety and efficacy of the bark.

## Data Availability Statement

All datasets generated for this study are included in the article/**Supplementary Material**.

## Author Contributions

RB and EP performed isolation by CPC. MS and CF-G performed LC-MS experiments and analysis. RB and SA performed quantitation by HPLC-PDA. JE performed the NMR analysis. SA and CF-G performed the enzymatic experiments and analysis. JR-P performed the docking studies. MS, JE, and EP wrote the manuscript.

## Funding

This research received funds from FONDECYT regular 1180059, Postdoctorado grants 3190794, and 3190572, and doctorado grant CONICYT PFCHA/beca doctorado nacional/2019-21191978. JE gratefully acknowledges funding from CONICYT(PAI/ACADEMIA N° 79160109).

## Conflict of Interest

The authors declare that the research was conducted in the absence of any commercial or financial relationships that could be construed as a potential conflict of interest.

## Abbreviations

AAPH, 2,2′-Azobis(2-amidinopropane) dihydrochloride; ABTS, 2,2′-Azino-bis(3-ethylbenzothiazoline-6-sulfonic acid); AChE, Acetylcholinesterase; ANOVA, One-way analysis of variance; ATCI, Acetyl-thiocholine iodide; AUC, Area under curve; BChE, Butyrylcholinesterase; BK, Bradykinin; BTC1, Butyryl-thiocholine chloride; CCC, Counter-current chromatography; COX, Cyclooxygenase; CPC, Centrifugal partition chromatography; CT, Cycle time; DPPH, 2,2,1-diphenyl-1-picrylhydrazyl; DTNB, 5-dithio-bis(2-nitrobenzoic) acid; ESI, Electrospray ionization; f3, Fluorescence reading at cycle 3; FL, Fluorescein disodium; F*n*, Fluorescence reading at cycle *n*; FRAP, Ferric Reducing Antioxidant Power; FWHM, Full width at half maximum; GAE, Gallic acid equivalent; h5-LOX, Human 5-lipoxygenase; HAT, Histone acetyltransferases; HEPES, (4-(2-hydroxyethyl)-1-piperazineethanesulfonic acid); HEMWAT, Hexane-ethyl acetate-methanol-water system; HSD, Honestly significant difference; IC_50_, Half maximal inhibitory concentration; ICAM-1, Intercellular adhesion molecule-1; ICH, International Council for Harmonization of Technical Requirements for Pharmaceuticals for Human Use; IL-1β, Interleukin-1β; IL-6, Interleukin-6; iNOS, Inducible Nitric Oxide Synthase; *k*, Partition coefficient; LOD, Limit of detection; LOQ, Limit of quantification; MAPK, mitogen-activated protein kinase; MMP, Matrix Metalloproteinase; MS, mass spectrometry; NF-*k*B, Nuclear factor kappa-light-chain-enhancer of activated B cells; NLRP3, NLR Family Pyrin Domain Containing 3; NO, Nitric oxide; ORAC, Oxygen radical absorbance capacity; OT, Orbitrap technology; PAF, Platelet activating factor; PBMC, Human peripheral blood mononuclear cells; PDA, Photodiode Array Detection; PTFE, Polytetrafluoroethylene; QE, Quercetin equivalent; ROS, Reactive oxygen species; RP, Reverse phase; RSD, Relative standard deviation; SD, Standard deviation; SP, Substance P; TE, Trolox equivalents; TEAC, Trolox equivalent antioxidant capacity; TFC, Total Flavonoid Content; TIC, Total Ion Chromatogram; TNF-α, Tumour Necrosis Factor alpha; TPC, Total Phenolic Content; TPTZ, 2,4,6-tri(2-pyridyl)-s-triazine; Trolox, 6-hydroxy-2,5,7,8-tetramethylchromane-2-carboxylic acid; UV, Ultraviolet; UHPLC, Ultra high performance chromatography; VCAM-1, Vascular cell adhesion molecule 1.
