# Supplementary material for: Metabolomic Analysis, Fast Isolation of Phenolic Compounds, and Evaluation of Biological Activities of the Bark From Weinmannia trichosperma Cav. (Cunoniaceae)
Source: Front Pharmacol. 2020 May 27;11:780. doi: 10.3389/fphar.2020.00780 (PMC7267059; doi:10.3389/fphar.2020.00780)
Supplement: Supplementary file 1 [file DataSheet_1.pdf]

Supplementary material

Metabolomic analysis, fast isolation of phenolic compounds, and evaluation of biological activities of the bark from *Weinmannia trichosperma* Cav. (Cunoniaceae)

Ruth Barrientos[1], Carlos Fernández-Galleguillos[1], Edgar Pastene[2], Mario Simirgiotis[1*], Javier Romero-Parra[3], Shakeel Ahmed[1], Javier Echeverría[4*]

[1] Instituto de Farmacia, Facultad de Ciencias, Universidad Austral de Chile, Valdivia, Chile.

[2] Laboratorio de Síntesis y Biotransformación de Productos Naturales, Departamento de Ciencias Básicas, Facultad de Ciencias, Universidad del Bío-Bío, Chillán, Chile.

[3] Departamento de Química Orgánica y Fisicoquímica, Facultad de Ciencias Químicas y Farmacéuticas, Universidad de Chile, Santiago, Chile.

[4] Departamento de Ciencias del Ambiente, Facultad de Química y Biología, Universidad de Santiago de Chile, Santiago, Chile.

*** Correspondence:**

Dr. Mario Simirgiotis, Instituto de Farmacia, Facultad de Ciencias, Universidad Austral de Chile, Valdivia, Chile, E-mail: mario.simirgiotis@uach.cl.

Dr. Javier Echeverria, Departamento de Ciencias del Ambiente, Facultad de Química y Biología, Universidad de Santiago de Chile, Santiago, Chile, E-mail: javier.echeverriam@usach.cl

HPLC Quantitative analysis

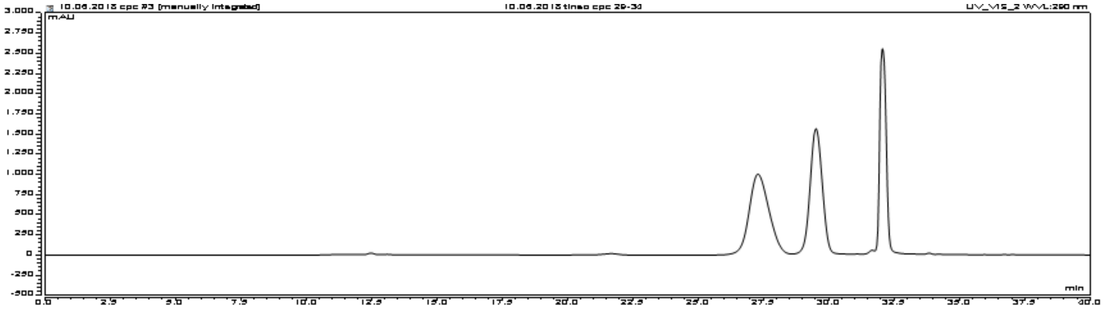

Figure S1. HPLC-PDA chromatogram of a tineo bark fraction showing three main isomers of astilbin.

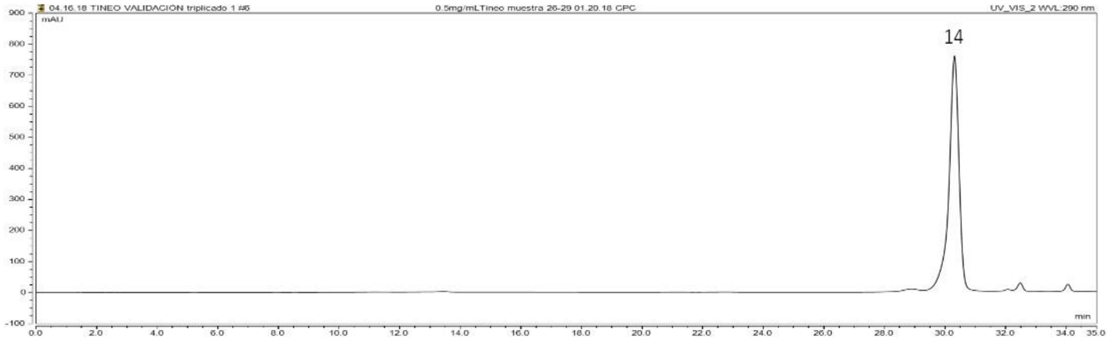

Figure S2. HPLC-PDA chromatogram of a CPC purified astilbin compound from tineo bark.

NMR analysis

NMR spectra of isoastilbin

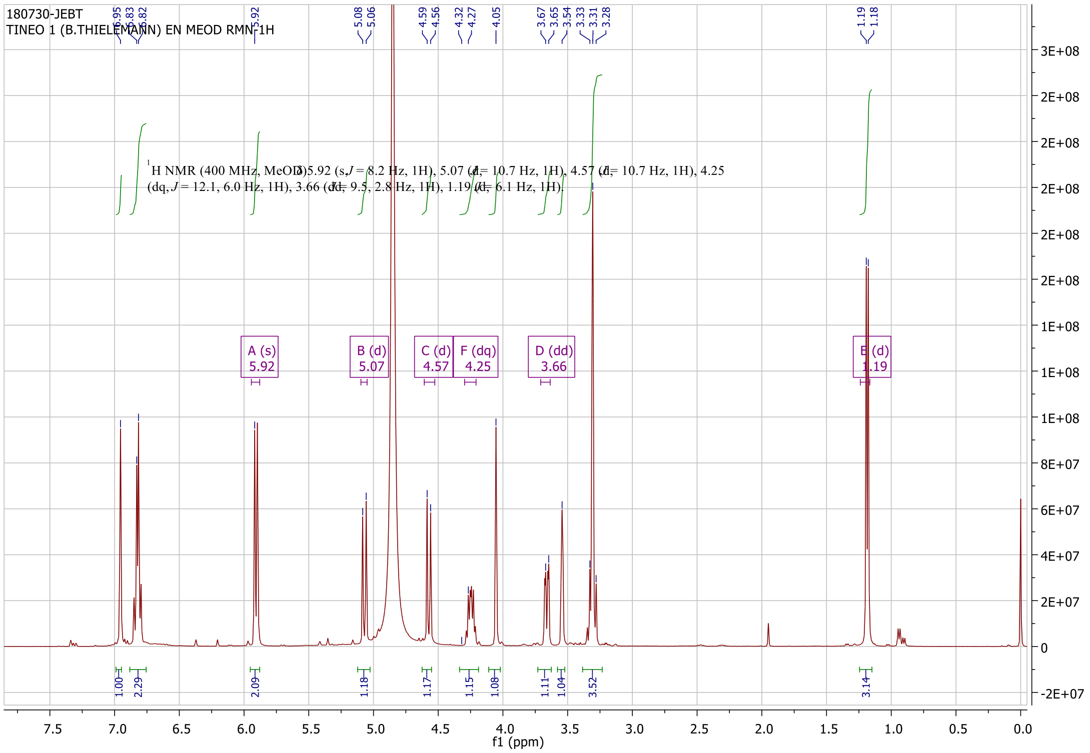

Figure S3. ^{1}H RMN spectra (400 MHz) for isoastilbin in MeOD.

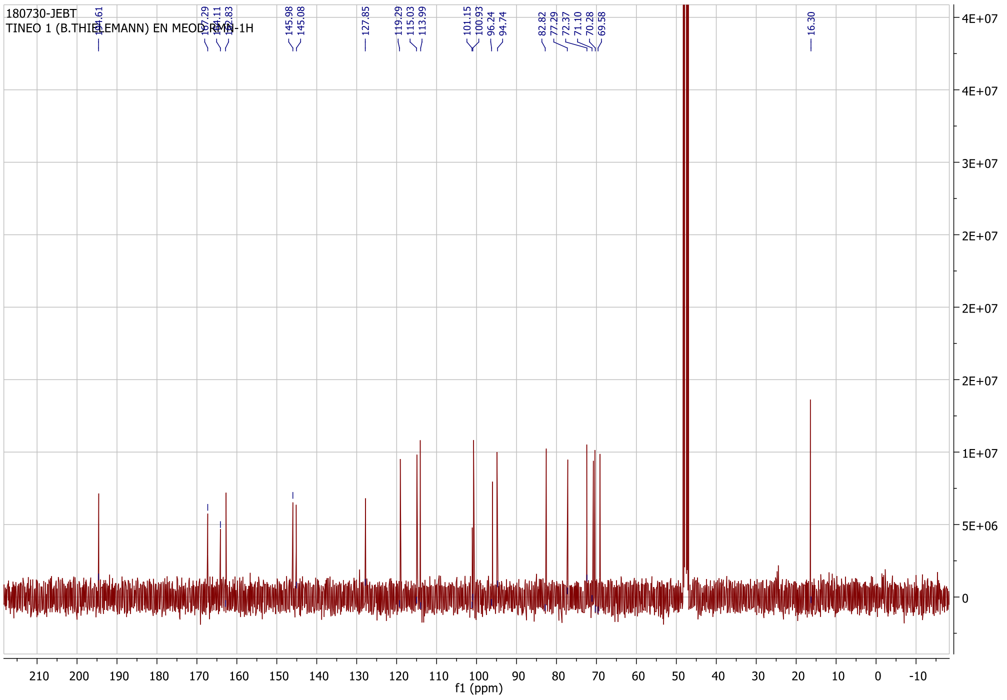

Figure S4. ^{13}C RMN spectra (125 MHz) for isoastilbin in MeOD

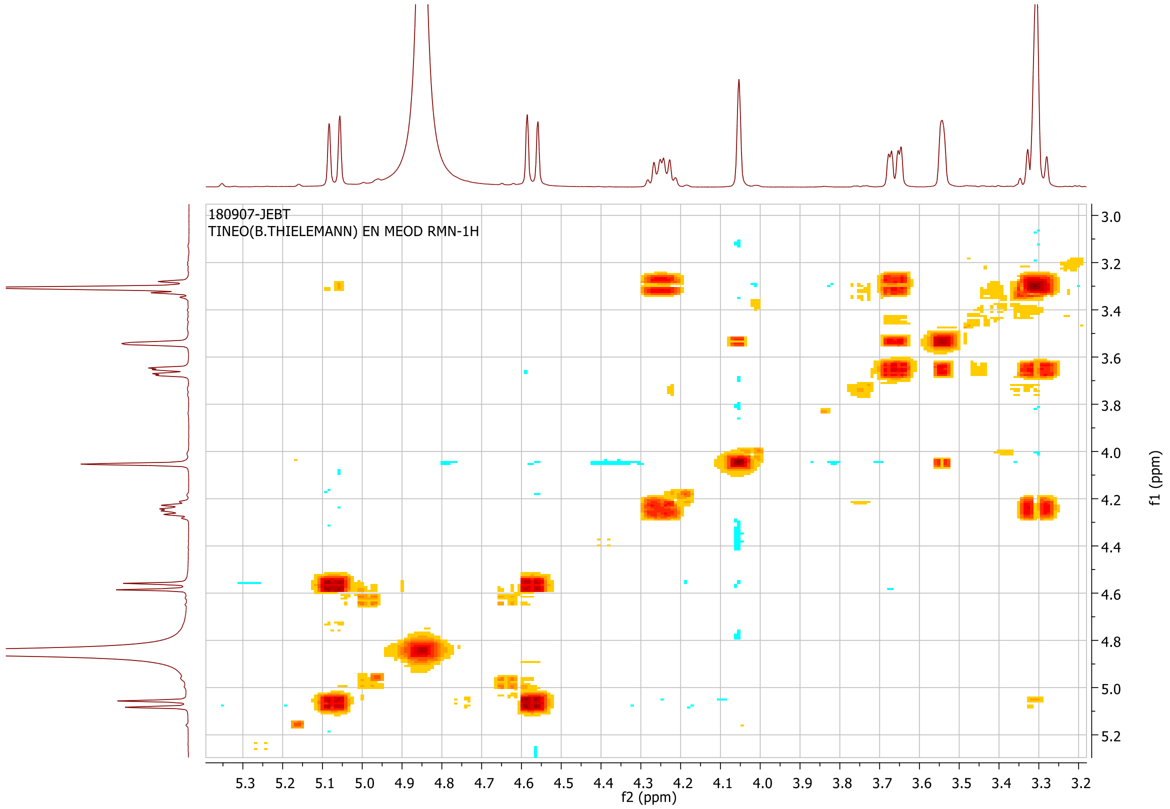

Figure S5. ^{1}H-^{1}H COSY spectra for isoastilbin.

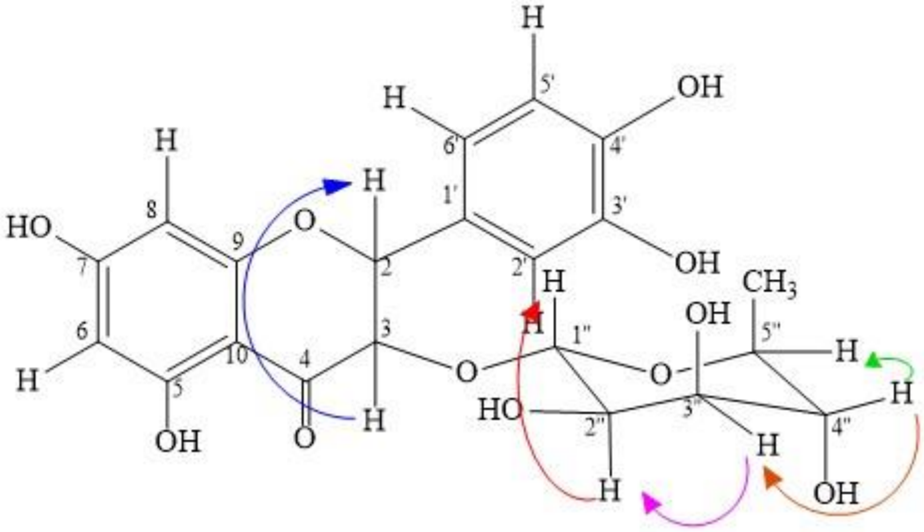

Figure S6. Important ^{1}H-^{1}H COSY correlations for isoastilbin.

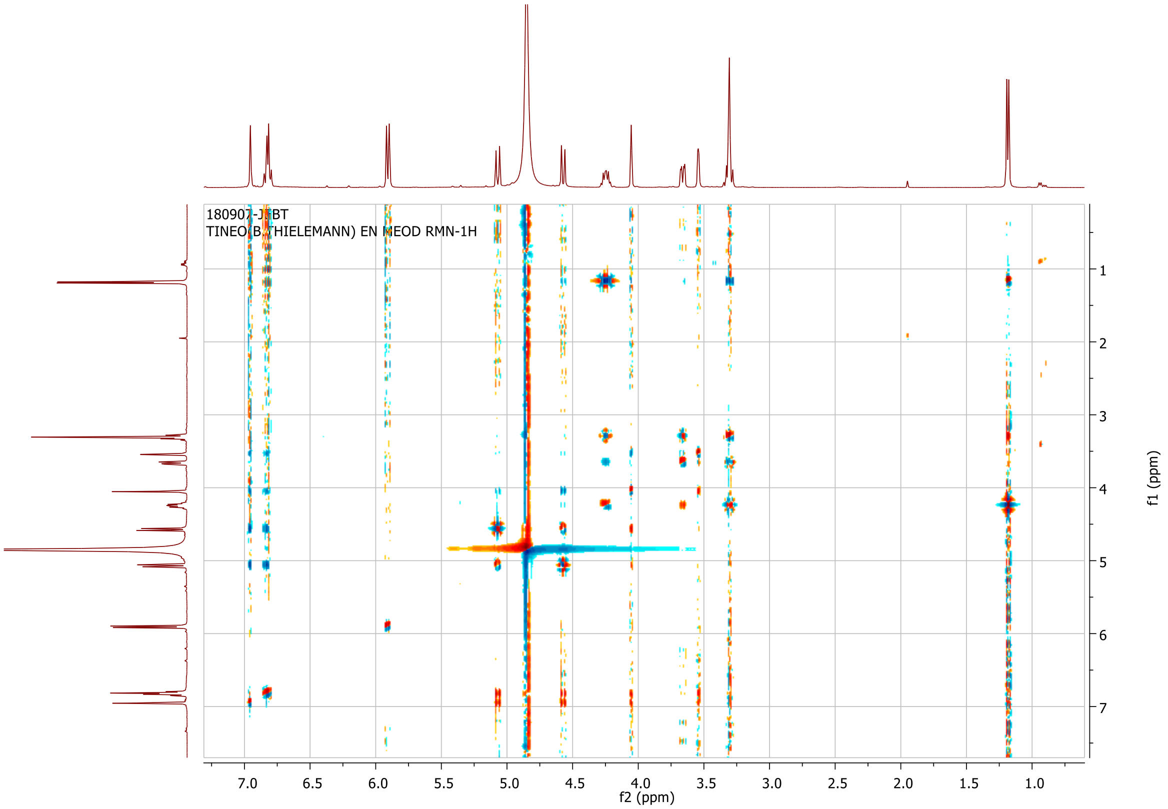

Figure S7. NOESY spectra for isoastilbin in MeOD

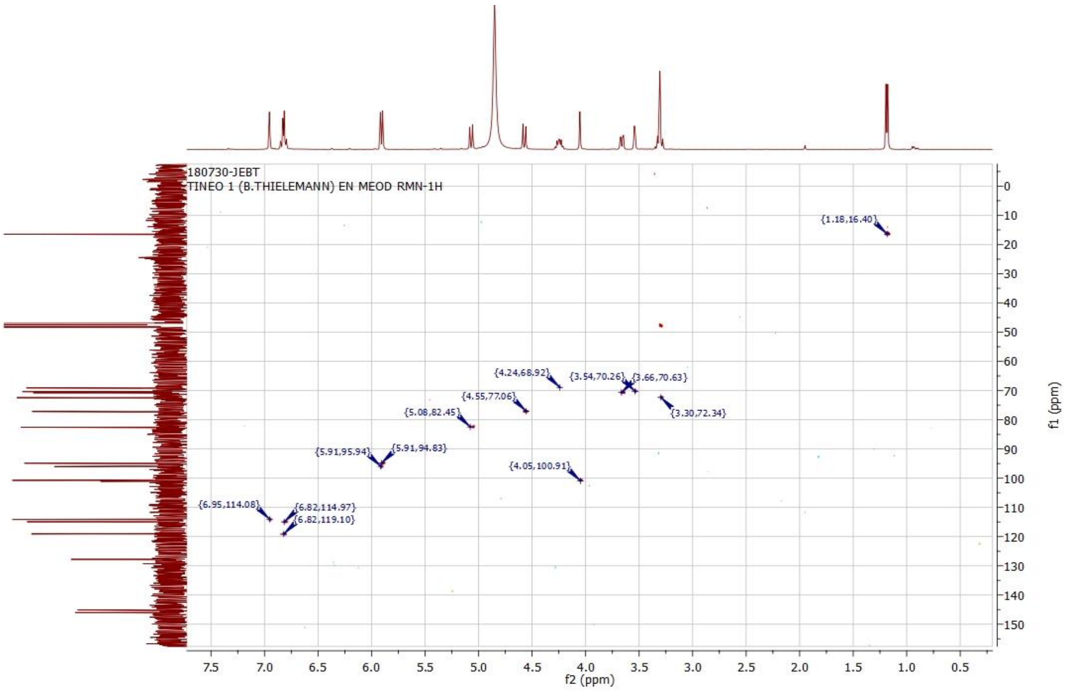

Figure S8. HMQC spectra for isoastilbin in MeOD

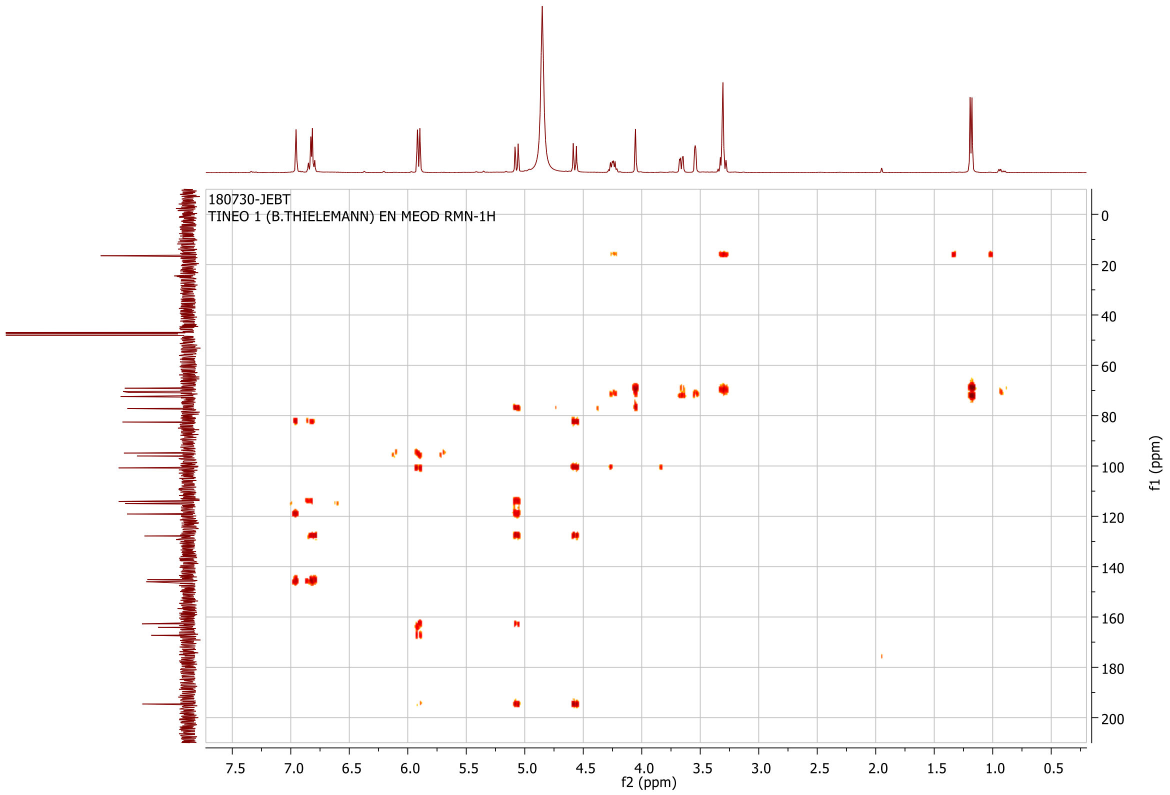

Figure S9. HMBC spectra for isoastilbin in MeOD

Full docking studies

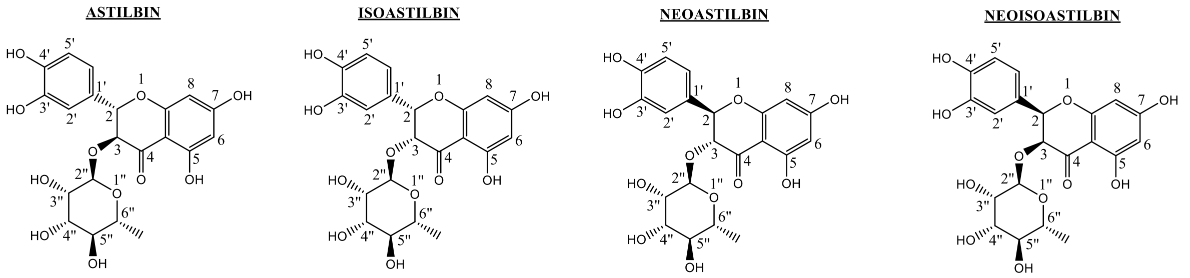

Figure S10. Isomers of astilbin

Material and Methods

Preparation of Astilbin, Isoastilbin, Neoastilbin, Neoisoastilbin, Galantamine and Zileuton

The geometries and partial charges of astilbin, isoastilbin, neoastilbin and neoisoastilbin contained in the *W. trichosperma* aqueous extract, as well as the known cholinesterases (*Tc*AChE- *h*BuChE) inhibitor galantamine and the 5-lipoxygenase (5- *h*LOX) inhibitor zileuton were fully optimised using the DFT method with the standard basis set PBE0/ 6-311+g*[1, 2]. All calculations were performed in Gaussian 09W software[3].

Molecular docking of isomers, Galantamine and Zileuton

Crystallographic enzyme structures of *Torpedo Californica* acetylcholinesterase (*Tc*AChE; PDBID: 1DX6 code[4]), human butyrylcholinesterase (*h*BuChE; PDBID: 4BDS code[5]) and 5-Lipoxygenase (5- *h*LOX; PDBID: 3V99 code[6]) were downloaded from the Protein Data Bank RCSB PDB[7]. Water molecules and ligands (in the case that they have been as a complex) of the crystallographic protein active sites were removed. All polar hydrogen atoms of each enzyme were added, and proteins were treated as rigid bodies. Grid maps were calculated using the autogrid option and were centred on the putative catalytic site of each enzyme considering their known catalytic residues: Ser200, Glu327 and His440 for *Tc*AChE[8, 9]; Ser198, Glu325 and His438 for *h*BuChE[10, 11]; and finally His367, His372, His550 (which are in charge of the coordination of the nonheme catalytic iron), as well as the main-chain carboxylate of the C-terminus Ile673 for 5-*h*LOX[6].

Ser200 of *Tc*AChE, Ser198 of *h*BuChE and the iron atom of 5-*h*LOX, whose importance lies in the fact that is involved in the transformation catalysis of Arachidonic Acid (AA) to the 5*S*-isomer of hydroperoxyeicosatetraenoic acid (5S-HPETE)[12, 13], were designated as the centre of the grids of the catalytic sites. The volumes chosen for the grid maps were made up of 60 × 60 × 60 points, with a grid-point spacing of 0.375 Å. Docked compound complexes were built using the Lamarckian Genetic Algorithm[14] which involved 100 runs. The lowest docked-energy binding cluster positions were chosen to be analyzed according to the potential intermolecular interactions between inhibitors and the enzymes, as well as to obtain the binding mode and docking descriptors. The different complexes were visualised in a Visual Molecular Dynamics program (VMD) and Pymol [15].

Results and Discussion

Acetylcholinesterase (TcAChE) docking results

Binding energy data of flavonoids astilbin, isoastilbin and neoastilbin obtained from docking assays over acetylcholinesterase shown in **Table 4**, exhibited similar values. This suggest that all these isomers possess similar ability to inhibit this enzyme. The similar binding energy values of these three compounds could explain why the experimental IC_{50} of the *W. trichosperma* aqueous extract and the IC_{50} of the isolated isoastilbin showed such close values (4.68$\pm$ 0.03 µg/mL and 3.13$\pm$ 0.32 µg/mL respectively). On the other hand, neoisoastilbin presented the best binding energy profile, which could lead to a higher inhibitory potency if this compound were isolated and tested as a single inhibitor. Therefore, since the *W. trichosperma* aqueous extract contain the four isomers (astilbin, isoastilbin, neoastilbin and neoisoastilbin) the eventual higher potency of neoisoastilbin compared to the other three isomers, we hypothesize that it cannot be detected due the existing competition among all flavonoids for the acetylcholinesterase catalytic site.

Astilbin also show three more Hydrogen bond interactions into the acetylcholinesterase cavity. These are carried out with Asp72, by the same hydroxyl group (-OH) that interacts with Asn85; with Ser200 through the hydroxyl group (-OH) at position 5- of the 5,7-dihydroxychroman-4-one core, and the last one between the hydroxyl (-OH) at position 7- of the 5,7-dihydroxychroman-4-one with Tyr130. Besides, this isomer performs an extra T-shaped interaction through the aromatic ring of His440 and the benzene moiety of the 5,7-dihydroxychroman-4-one (**Figure S11A**).

Isoastilbin, in addition to the interactions with Asn85, Tyr121 and Glu199, also showed one more Hydrogen bond interaction between one of the hydroxyl groups (-OH) of the dihydroxyphenyl core with Tyr70, as well as a π-π interaction through the dihydroxyphenyl core and Trp84 residue (**Figure S11B**).

Neoastilbin and neoisoastilbin presented different binding modes into the acetylcholinesterase compared to astilbin and isoastilbin. In the same manner, these two compounds also showed in concordance poses into the catalytic site between themselves. It can be seen from **Figure S11C** that neoastilbin show several Hydrogen bond interactions with Asp72, Tyr121, Ser122 and Glu199, as well as a π-π interaction between the benzene core of the 5,7-dihydroxychroman-4-one and Tyr121. On the other hand, neoisoastilbin exhibited a docking pose which resulted in the execution of even more Hydrogen bond interactions compared with all the other isomers. This could explain its favourable binding energy of -10.37 kcal/mol. The Hydrogen bond interactions performed by neoisoastilbin involve many amino acids, such as Asp72, Tyr121, Glu199, Ser200, Phe330 and His440. Furthermore, neoisoastilbin performed a π-π interaction through the dihydroxyphenyl ring and the aromatic moiety of Tyr121 (**Figure S11D**).

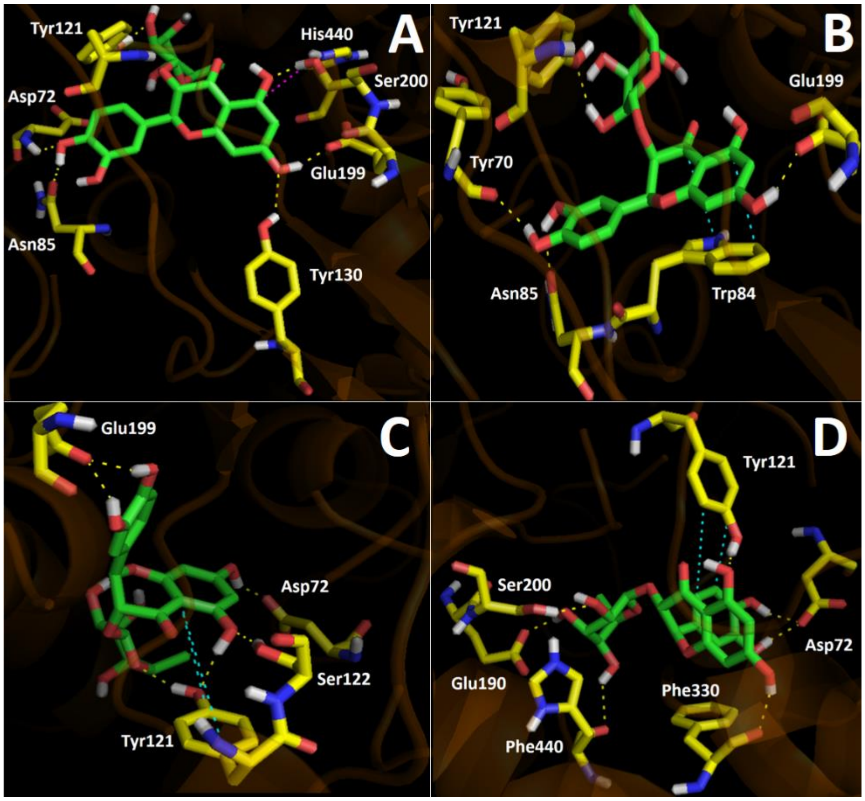

Figure S11. Predicted binding mode and predicted intermolecular interactions among all flavonoids and the residues of *Torpedo Californica* acetylcholinesterase (*Tc*AChE) catalytic site. Yellow dotted lines indicate Hydrogen bond interactions, magenta dotted lines indicates T-Shaped interactions, and cyan dotted lines represents π-π interactions. **A.** Astilbin into the catalytic site (six H-bondings with Asp72, Asn85, Tyr121, Tyr130, Glu199 and Ser200; T-Shaped with His440). **B.** Isoastilbin into the catalytic site (four H-bondings with Tyr70, Asn85, Tyr121 and Glu199; one π-π interaction with Trp84). **C.** Neoastilbin into the catalytic site (six H-bondings with Asp72, Tyr121, Ser122 and Glu199; one π-π interaction with Tyr121). **D.** Neoisoastilbin into the catalytic site (seven H-bondings with Asp72, Tyr121, Glu199, Ser200, Phe330 and His440; one π-π interaction with Tyr121).

Butyrylcholinesterase (hBuChE) docking results

Binding energies of all isomers obtained in docking assays over butyrylcholinesterase (*h*BuChE) also showed similar values among themselves, even when they are compared with the energy obtained for the cholinesterases inhibitor galantamine. The latter correlates with our enzymatic inhibition assays, where the IC_{50} value for isolated isoastilbin was 2.94± 0.08 µg/mL, which is similar to galantamine IC_{50}, value suggesting an analogous ability to inhibit the butyrylcholinesterase. As in the case of acetylcholinesterase, the fact that the IC_{50} value of the *W. trichosperma* aqueous extract (8.51± 0.03 µg/mL) was higher than isolated isoastilbin could be attributed to the competition among all isomers contained in the extract for the butyrylcholinesterase catalytic site.

Docking analysis showed that astilbin and isoastilbin share relative similarity binding modes into the catalytic site of the enzyme, projecting their 5,7-dihydroxychroman-4-one, dihydroxyphenyl and glycoside frameworks towards the same directions into the cavity of the butyrylcholinesterase. Likewise, neoastilbin and neoisoastilbin also showed a similar overlap and projecting behavior between themselves. As a matter of fact, the couple astilbin/isoastilbin, as well as the couple neoastilbin/neoisoastilbin, performed some Hydrogen bond interactions with the same amino acid residues into the catalytic site. In the case of astilbin and isoastilbin both isomers carried out Hydrogen bond interactions through their hydroxyl (-OH) groups at position 5 of the 5,7-dihydroxychroman-4-one core and the amino acid Asp70, with the particularity that in astilbin the oxygen atom of this hydroxyl group (-OH) participates in the interaction with the hydrogen atom of the amide from the peptide bond (**Figure S12A**), while in isoastilbin the hydrogen atom of this same hydroxyl group (-OH) is in charge to perform the Hydrogen bonding. Moreover, these both isomers share another Hydrogen bond interaction with the amino acid Glu197 through the hydroxyl entities (-OH) of their dihydroxyphenyl moieties (**Figure S12A and S12B**).

Other main interactions showed by astilbin into the catalytic site of butyrylcholinesterase enzyme are those carried out with the residues of Gln67 and Tyr332, which with those already mentioned above are responsible for the similar Galantamine binding energy.

Concerning the isoastilbin isomer, this compound also exhibited Hydrogen bond interactions with Asn83, Trp430 and Tyr440. Moreover, the dihydroxyphenyl core of this molecule perform a π-π interaction with Trp82 (**Figure S12B**). Although, isoastilbin exhibit one extra π-π interaction that Astilbin lacks. This latter isomer possesses a better binding energy profile as can be seen in **Table 4**, which should not represent a great relevance in terms of enzymatic inhibition.

As was already mentioned, neoastilbin and neoisoastilbin are arranged in a similar manner into the catalytic cavity of butyrylcholinesterase, directing their dihydroxyphenyl and glycoside frameworks towards the same spots. Therefore, they share some interactions with the same amino acids, such as Hydrogen bondings between the hydroxyl groups (-OH) of their dihydroxyphenyl cores with Gly117, and another Hydrogen bond interaction between the hydroxyl groups (-OH) of their glycoside cores with Tyr440. Considering all interactions that neoastilbin carried out, we can find six Hydrogen bonds with Gly116, Gly117, Tyr128, Glu197, Trp430, Tyr440, and one π-π interaction through the benzene moiety of the 5,7-dihydroxychroman-4-one and the aromatic ring of Phe329 (**Figure S12C**).

Neoisoastilbin interacts in the same manner that the other inhibitors do performing five Hydrogen bond interactions with Trp82, Gly115, Gly116, Gly117, and Tyr440, as well as, one π-π interaction through the 5,7-dihydroxychroman-4-one and the aromatic ring of Trp82. All these chemical properties for neoisoastilbin results in the lowest binding energy value for all butyrylcholinesterase isomers evaluated in our docking assays.

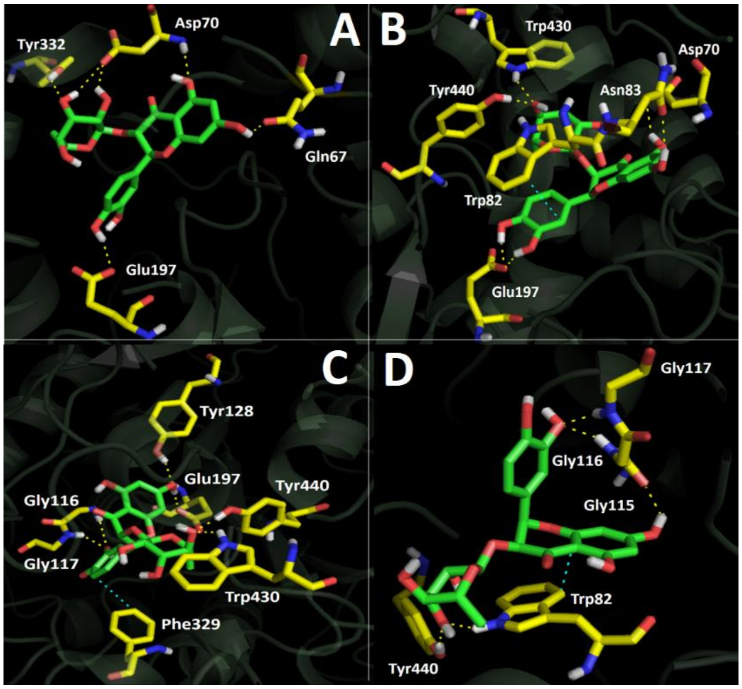

Figure S12. Predicted binding mode and predicted intermolecular interactions among all flavonoids and the residues of human butyrylcholinesterase (*h*BuChE) catalytic site. Yellow dotted lines indicate Hydrogen bond interactions and cyan dotted lines represents π-π interactions. **A.** Astilbin into the catalytic site (six H-bondings with Gln67, Asp70, Tyr332 and Glu197; no T-Shaped or π-π interaction performed). **B.** Isoastilbin into the catalytic site (six H-bondings with Asp70, Asn83, Glu197, Trp430 and Tyr440; one π-π interaction with Trp82). **C.** Neoastilbin into the catalytic site (six H-bondings with Gly 116, Gly117, Tyr128, Glu197, Trp430 and Tyr440; one π-π interaction with Phe329). **D.** Neoisoastilbin into the catalytic site (five H-bondings with Trp82, Gly115, Gly116, Gly117 and Tyr 440; one π-π interaction with Trp82).

5-Lipoxygenase (5- hLOX) docking results

Binding energy values obtained over 5-lipoxygenase (5-*h*LOX) by astilbin, isoastilbin, neoastilbin and neoisoastilbin showed to be not that well as the energies achieved for these same compounds over acetylcholinesterase (*Tc*AChE) and butyrylcholinesterase (*h*BuChE), especially when they are compared to the binding energy of the known 5-Lipoxygenase inhibitor Zileuton (Table **4**). These docking results are in agreement with our enzymatic inhibition experiments obtained over the 5-*h*LOX, where the enzymatic inhibition percentage for the *W. trichosperma* aqueous extract was 82.86%, and for the isolated isoastilbin was 34.29% at 10 µM and 80.71% at 40 µM. Docking assays revealed that all isomers performed only two Hydrogen bonds interactions at the most. No one of them exhibited π-π interactions or T-shaped interactions. Additionally, no other type of contributing non-covalent bonds, such as salt bridges and π-cation interaction were observed. The latter could be explained due that the studied flavonoids structures lack of ionizable groups which could interact with the enzyme increasing the affinity, and therefore, the enzymatic inhibition.

Once again astilbin and isoastilbin into the catalytic site of the 5-*h*LOX resulted to be superimposed. Thus, both derivatives showed two Hydrogen bond interactions with the same amino acid residues. The first one, through an oxygen atom of one of the hydroxyl groups (-OH) of the glycoside nucleus with Tyr181; and the second, through an oxygen atom of one of the hydroxyl groups (-OH) of the dihydroxyphenyl core with Thr364 (**Figure S13A and S13B).**

Docking experiments over 5-*h*LOX of neoastilbin and neoisoastilbin showed different poses patterns into the catalytic site of the 5-Lipoxigenase. Therefore, both neoastilbin and neoisoastilbin carried out Hydrogen bond interactions with different amino acids and through different hydroxyl groups (-OH) of their structures. Neoastilbin performed a Hydrogen bond interaction with His600 through an oxygen atom of one of its Hydroxyl groups (-OH) of the dihydroxyphenyl framework, as well as a Hydrogen bond interaction between the oxygen atom of the hydroxyl function (-OH) at position 5- of the 5,7-dihydroxychroman-4-one and the hydrogen atom of heterocyclic ring of His367 (**Figure S13C**). On the other hand, as can be seen in **Figure S13D**, neoisoastilbin, which is the worst inhibitor in terms of binding energy, shows two Hydrogen bond interactions. The first one between Asn425 and the hydroxyl group (-OH) at position 5- of the 5,7-dihydroxychroman-4-one moiety, and the second one between an oxygen atom of one of the hydroxyl group (-OH) of the dihydroxyphenyl framework at position 2- of the 5,7-dihydroxychroman-4-one nucleus.

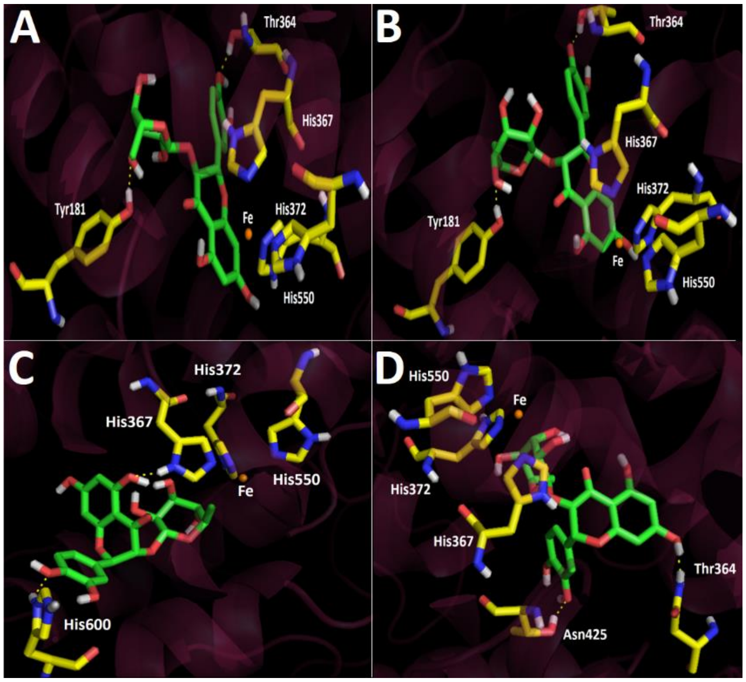

Figure S13. Predicted binding mode and predicted intermolecular interactions among all flavonoids and the residues of human 5-Lipoxigenase (5-*h*LOX) catalytic site. Yellow dotted lines indicate Hydrogen bond interactions. **A.** Astilbin into the catalytic site (two H-bondings with Tyr181 and Thr364). **B.** Isoastilbin into the catalytic site (two H-bondings with Tyr181 and Thr364). **C.** Neoastilbin into the catalytic site (two H-bondings with His367 and His600). **D.** Neoisoastilbin into the catalytic site (two H-bondings with Thr364 and Asn425).

Quantification of astilbin isomers

Table S1. Content of isoastilbin isomers in infusion and ethanol extract of *W. trichosperma* (mg/g of extract).

Compounds	Infusion extract	Tincture extract
Isoastilbin	141,7366 ± 1,1872	137.8546 ± 1.2281
Neoastilbin	99.5973 ± 0.8376	95.3789 ± 0.7896
Neoisoastilbin	53.1795 ± 0.3191	51.1567 ± 0.31187

Table S2. Analytical parameters and validation results for linearity, LOQ, LOD, accuracy and precision of UHPLC-PDA method for astilbin isomers.

Compound	R_t (min)	Linearity (r^2)	Intercept	Slope	LOQ	LOD	Repeatability of calibration curve
Neoastilbin	18.0	0.9975	-1.569	547.22	21	8	0.5141
Isoastilbin	29.3	0.99568	-2.4065	571.63	20	6	0.5849
Neoisoastilbin	31.5	0.9949	-1.6702	574.53	20	8	0.5233

Table S3. Analytical parameters and validation results for linearity, LOQ, LOD, accuracy and precision of UHPLC-PDA method for isoastilbin

Parameter	
Linearity range (mg/mL)	0.01-0.5
Determination coefficient (r^2)	0.99568
Intercept	-2.4065
Slope	571.6340
Linear regression equation	$y = 571.6340x – 2.4065$
Limit of detection (LOD) (ng)	6
Limit of quantification (LOQ) (ng)	20
Retention time (min)	29.3

Table S4. Repeatability of calibration curve and intermediate precision for isoastilbin.

Concentración (μg/mL)	Repeatability		Intermediate precision	
	Mean AUC (mAu*min)	RSD (%)	Mean AUC (mAu*min)	RSD (%)
0.01	5.3477	1.3006	4.5839	2.4872
0.03	16.056	0.58491	-	-
0.05	27.093	2.5509	28.170	0.48978
0.1	54.854	0.87662	-	-
0.3	152.81	1.9153	-	-
0.5	293.48	1.1042	266.63	0.36527

Table S5. Recovery studies of isoastilbin.

Concentration (mg/mL)	Mean of interpolated concentration ± SD	% Recovery
**0.01**	0.01223 ± 0.00020	113.8
**0.05**	0.05349 ± 0.00024	88.23
**0.5**	0.47065 ± 0.00170	94.6

Table S6. Absolute recovery of isoastilbin in aqueous extract of *W. trichosperma*.

Fortification (%)	Mean AUC ± SD (mAu*min)	RSD (%)	% Recovery	mg isoastilbin/g extract
0	0.031225 ± 0.000166	0.53204	-	25.81
30	0.040276 ± 0.000249	0.61905	128.99	33.29
50	0.044417 ± 0.000107	0.24285	142.25	36.71

References

1. Adamo, C. and V. Barone. (1990). Toward reliable density functional methods without adjustable parameters: The PBE0 model. *J. Chem. Phys.* 110(13): 6158-6170. doi: 10.1063/1.478522
2. Petersson, A., Bennett, A., Tensfeldt, T. G., Al-Laham, M. A., Shirley, W. A., and Mantzaris, J. (1988). A complete basis set model chemistry. I. The total energies of closed-shell atoms and hydrides of the first-row elements. *J. Chem. Phys.* **89**(4): 2193-2218. doi: 10.1063/1.455064
3. Frisch, A. (2009). *Gaussian 09 User's Reference*. Gaussian, Inc.
4. Greenblatt, H. M., Kryger, G., Lewis, T., Silman, I., and Sussman, J. L. (1999). Structure of acetylcholinesterase complexed with (−)-galanthamine at 2.3 Å resolution. *FEBS Lett.* 463(3): 321-326. doi: 10.1016/S0014-5793(99)01637-3
5. Nachon, F., Carletti, E., Ronco, C., Trovaslet, M., Nicolet, Y., Jean, L., and Renard, P. Y. (2013). Crystal structures of human cholinesterases in complex with huprine W and tacrine: elements of specificity for anti-Alzheimer's drugs targeting acetyl-and butyryl-cholinesterase. *Biochem. J.* 453(3): 393-399. doi: 10.1042/BJ20130013
6. Gilbert, N. C., Bartlett, S. G., Waight, M. T., Neau, D. B., Boeglin, W. E., Brash, A. R., and Newcomer, M. E. (2011). The structure of human 5-lipoxygenase. *Science* **331**(6014): 217-219. doi: 10.1126/science.1197203
7. Berman, H. M., Westbrook, J., Feng, Z., Gilliland, G., Bhat, T. N., Weissig, H., *et al*. (2000) RCSB Protein Data Bank: Structural biology views for basic and applied research. *Nucleic Acids Res*. 28: 235-242.
8. Silman, I., Harel, M., Axelsen, P., Raves, M., and Sussman, J. L. (1994). Three-dimensional structures of acetylcholinesterase and of its complexes with anticholinesterase agents. *Biochem. Soc. Trans.* 22(3): 745-749.
9. Sussman, J. L., Harel, M., Frolow, F., Oefner, C., Goldman, A., Toker, L., & Silman, I. (1991). Atomic structure of acetylcholinesterase from Torpedo californica: a prototypic acetylcholine-binding protein. *Science* 253(5022): 872-879. doi: 10.1126/science.1678899
10. Nicolet, Y., Lockridge, O., Masson, P., Fontecilla-Camps, J. C., and Nachon, F. (2003). Crystal structure of human butyrylcholinesterase and of its complexes with substrate and products. *J. Biol. Chem.* 278(42): 41141-41147. doi: 10.1074/jbc.M210241200
11. Tallini, L. R., Bastida, J., Cortes, N., Osorio, E. H., Theoduloz, C., & Schmeda-Hirschmann, G. (2018). Cholinesterase Inhibition Activity, Alkaloid Profiling and Molecular Docking of Chilean Rhodophiala (Amaryllidaceae). *Molecules* 23(7): 1532. doi: 10.3390/molecules23071532
12. Rådmark, O. and B. Samuelsson (2009). 5-Lipoxygenase: mechanisms of regulation. *J Lipid Res*. 50(Supplement): S40-S45. doi: 10.1194/jlr.R800062-JLR200
13. Shimizu, T., Rådmark, O., & Samuelsson, B. (1984). Enzyme with dual lipoxygenase activities catalyzes leukotriene A4 synthesis from arachidonic acid. *Proc. Natl. Acad. Sci. U. S. A.* 81(3): 689-693. doi: 10.1073/pnas.81.3.689
14. Thomsen, R., and Christensen, M. H. (2006). MolDock: a new technique for high-accuracy molecular docking. *J. Med. Chem.* 49(11): 3315-3321. doi: 10.1021/jm051197e
15. DeLano, W.L. (2002) *The PyMOL molecular graphics system.* http://www.pymol.org.
